# Genome-wide association analysis and KASP markers development for protein quality traits in winter wheat

**DOI:** 10.1186/s12870-025-06171-z

**Published:** 2025-02-05

**Authors:** Yousheng Tian, Pengpeng Liu, Dezhen Kong, Yingbin Nie, Hongjun Xu, Xinnian Han, Wei Sang, Weihua Li

**Affiliations:** 1 Cotton Research Institute, Xinjiang Academy of Agricultural and Reclamation Sciences, Shihezi, 832000 China; 2 The Key Laboratory of the Oasis Ecological Agriculture, College of Agriculture, Shihezi University, Shihezi, 832003 China; 3 Institute of Crop Science, Xinjiang Academy of Agricultural and Reclamation Sciences, Shihezi, 832000 China; 4 Key Laboratory of Xinjiang Production and Construction Corps for Cereal Quality Research and Genetic Improvement, Xinjiang Academy of Agricultural and Reclamation Sciences, Shihezi, 832000 China

**Keywords:** Wheat, Protein quality, GWAS, KASP, Candidate genes

## Abstract

**Background:**

Wheat (*Triticum aestivum* L.) is a significant cereal crop that plays a vital role in global food production. To expedite the breeding of wheat cultivars with high protein quality, it is necessary to genetically analyze the traits related to quality. A genome-wide association study (GWAS) was conducted to identify the genomic regions responsible for protein quality traits in winter wheat.

**Results:**

Six protein quality traits were evaluated across two locations and two years for a total of 341 wheat accessions. Utilizing the wheat 40 K SNP array, GWAS identified 97 significantly stable SNPs at 43 loci for five out of six protein quality traits using a linear mixed model. The 43 loci distribution was four for grain protein content, two for flour protein content, one for wet gluten content, four for gluten index, and thirty-two for Zeleny sedimentation value. The most significant associations were identified on chromosomes 1 A, 1B, and 1D. Haplotype analysis of loci associated with the gluten index in the 412–416 Mb interval on chromosome 1D identified three blocks. Accessions with superior haplotypes showed a significantly higher gluten index than those with inferior haplotypes. Six KASP markers were successfully developed for the gluten index, while five KASP markers were developed for the Zeleny sedimentation value. Additionally, eight candidate genes were identified that may affect protein accumulation during grain development.

**Conclusions:**

Our study identified 97 SNPs significantly associated with protein quality traits; developed 6 KASP markers for gluten index, and 5 KASP markers for Zeleny sedimentation values; screened 8 candidate genes that may be related to protein quality during grain development. Thise research will offer valuable insights for wheat breeding programs in China and globally.

**Supplementary Information:**

The online version contains supplementary material available at 10.1186/s12870-025-06171-z.

## Background

Wheat (*Triticum aestivum* L.) is a widely adapted and important food crop [1], supplying an average of 20% of the total calories and 22% of the total protein in the human diet [2]. Protein is a primary quality component of cereal grains. Protein content and quality have been shown to affect one or more attributes of noodle quality [3, 4], also play an important role in baking performance [5] and bread and pasta quality [6]. Gluten proteins (∼80%) are the major storage proteins that impact the baking process through flour’s functional properties [7]. Zeleny sedimentation value has been used as a critical trait in detecting gluten strength as well as in estimating the quality of wheat as a diet and an ingredient in cooking [8, 9]. Recently, the increasing consumer demand for food with higher nutritional value has led breeding programs to shift their focus towards improving grain quality, in addition to achieving high productivity [10].

Grain protein quality is a complex trait determined by multiple quantitative trait loci (QTL) that interact with each other and with the environment [11]. The protein quality profile of wheat lines should be assessed within breeding programs [12]. However, protein quality tests are highly resource-intensive and almost impossible to conduct in the early generations of the breeding program [13]. What’s more, assessing wheat protein quality is often costly and time-consuming [14]. Marker-assisted selection (MAS) can be beneficial for selecting these quality traits. In MAS approaches, genetic loci must first be identified, and linked or diagnostic markers must be developed [15].

Genome-wide association study (GWAS) is a useful technique with more accurate results because of having more genetic diversity and historical recombination of alleles in associated panels [16]. Recently, GWAS has been used to analyze various complex traits in plants [17, 18]. Molecular marker technology is continuously improving, providing higher genotypic resolution while reducing time and costs [19]. Kompetitive allele-specific PCR (KASP) is a leading SNP genotyping technology [20], which uses endpoint fluorescence detection to discriminate tagged alleles [21]. Since the advent of KASP, it has been widely utilized in various crops, including wheat, rice, maize, soybean, cucumber, and others [22–28].

Many quantitative trait loci (QTL) controlling protein quality traits have been located on all wheat chromosomes in several previous studies [29–33]. The majority of QTLs for grain protein content and flour protein content were reported on chromosomes 6B and 7B. The marker *Gpc-B1* on chromosome 6B has been utilized in wheat quality breeding [34]. Major loci controlling glutenin and gliadin have been mapped to homoeologous chromosome groups 1 and 6 [35]. Two major QTL clusters for glutenin were reported on 1DL-2 and 6AS-3, in the chromosome regions 409.26–416.45 Mb and 44.02–50.35 Mb, respectively [36]. Pleiotropic loci (*BS00063551_51* and *RAC875_c28721_290*) associated with grain protein content and gluten content were identified on chromosomes 1B and 3 A, respectively, through GWAS using a 90 K single nucleotide polymorphism (SNP) array in bread wheat [37]. The association marker *SNP_1025300* for Zeleny sedimentation value was identified on chromosome 1 A through GWAS using both the MLM and FarmCPU models in 802 spring bread wheat breeding lines [38]. However, many of these QTLs are specific to certain populations or environments, making it difficult to apply them in our breeding programs [39]. Herein, we performed a GWAS to identify genetic loci associated with grain protein quality traits using the 40 K SNP assay and two years of protein quality trait data from a panel of 341 winter wheat genotypes. The KASP marker was further developed for the identified significant MTAs. Our study will provide valuable insights for wheat breeding programs in China and globally.

## Results

### Phenotypic assessment

The analysis of variance (ANOVA) for the association panel showed highly significant variations in all traits across all environments (Table S6). The coefficients of variation for grain protein content, flour protein content, wet gluten content, dry gluten content, gluten index, and Zeleny sedimentation value ranged from 4.73 to 6.60%, 4.25–6.68%, 10.96–11.64%, 10.54–12.68%, 17.14–31.88%, and 11.08–14.37%, respectively, across the different environments. The *h*^2^ estimates for these traits were 0.83, 0.78, 0.76, 0.74, 0.67, and 0.87, respectively (Table 1). The frequency distributions of BLUP values for protein quality traits were all nearly symmetrically distributed (Fig. S1). Correlation analysis was conducted among the protein quality traits based on BLUP values across four environments (Table 2). Except for the gluten index, which showed a significant negative correlation with grain protein content, flour protein content, wet gluten content, and dry gluten content (−0.086**, −0.056*, −0.265**, and − 0.179**, respectively), there were significant positive correlations among the protein quality traits, with the highest correlation was observed between dry gluten content and wet gluten content (0.977**).

**Table 1:** Phenotypic variations and heritability of protein quality traits

Trait	Environment	Minimum	Maximum	Mean	SD	CV (%)	*h*^*2*^
Grain protein content	2020_EM	13.80	19.00	16.16	1.00	6.20	0.83
	2020_QT	12.00	16.80	14.03	0.92	6.59	
	2021_EM	16.10	21.10	18.46	0.87	4.73	
	2021_QT	13.10	19.00	15.89	1.05	6.60	
Flour protein content	2020_EM	13.00	17.70	15.29	0.88	5.78	0.78
	2020_QT	11.00	15.60	13.34	0.89	6.68	
	2021_EM	15.50	19.50	17.30	0.73	4.25	
	2021_QT	12.40	18.05	15.01	1.00	6.66	
Wet gluten content	2020_EM	29.00	54.30	41.89	4.59	10.96	0.76
	2020_QT	27.60	50.10	37.95	4.23	11.15	
	2021_EM	37.65	72.10	53.11	5.99	11.28	
	2021_QT	32.15	58.50	43.73	5.09	11.64	
Dry gluten content	2020_EM	11.50	20.54	15.75	1.66	10.54	0.74
	2020_QT	10.03	18.88	14.08	1.66	11.81	
	2021_EM	15.30	29.74	20.73	2.62	12.63	
	2021_QT	11.82	22.53	16.51	2.09	12.68	
Gluten index	2020_EM	4.71	98.45	60.43	19.27	31.88	0.67
	2020_QT	19.10	100.00	62.71	14.29	22.79	
	2021_EM	27.43	97.06	62.06	10.63	17.14	
	2021_QT	23.64	87.91	54.50	10.28	18.86	
Zeleny sedimentation value	2020_EM	21.00	47.50	34.35	4.68	13.61	0.87
	2020_QT	20.00	44.00	31.48	4.53	14.37	
	2021_EM	25.50	50.00	36.87	4.08	11.08	
	2021_QT	17.00	44.00	30.30	4.44	14.66	

2020_EM, 2020_QT, 2021_EM, and 2021_QT represent the cropping seasons of 2019–2020 and 2020–2021 in E’min (EM) and Qitai (QT) respectively. SD stands for standard deviation. CV stands for coefficient of variation. *h*^2^ represents heritability, which was generated across four different environments.

**Table 2:** Phenotypic correlations (r) of the protein parameters for the winter wheat association mapping panel

Trait	Grain protein content	Flour protein content	Wet gluten content	Dry gluten content	Gluten index
Flour protein content	0.972^**^				
Wet gluten content	0.848^**^	0.844^**^			
Dry gluten content	0.840^**^	0.840^**^	0.977^**^		
Gluten index	−0.086^**^	−0.056^*^	−0.265^**^	−0.179^**^	
Zeleny sedimentation value	0.416^**^	0.443^**^	0.203^**^	0.234^**^	0.470^**^

** indicates significant differences at *P* < 0.01

### Structure analysis

**Fig. 1:**
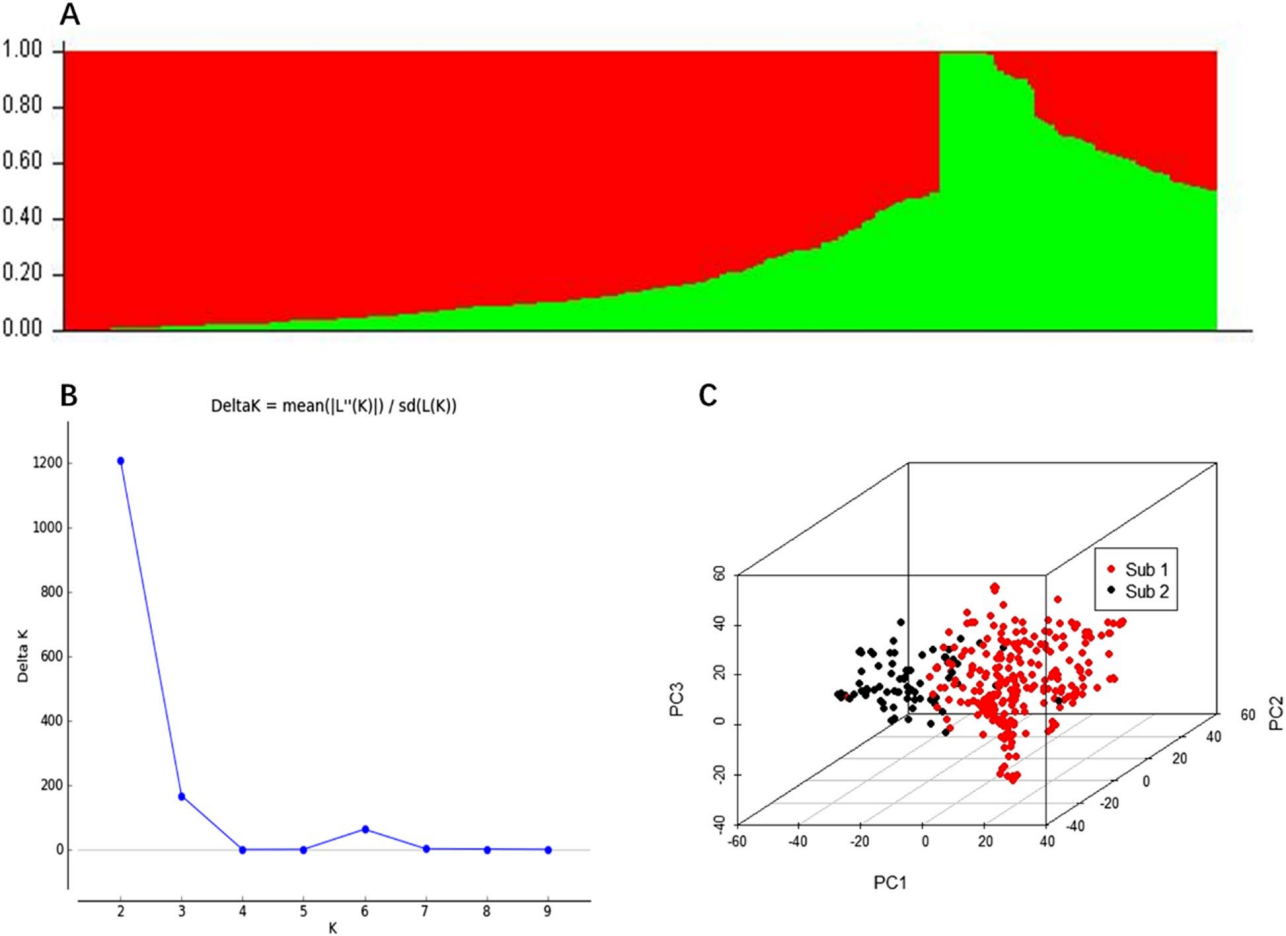
Population structure of 341 wheat accessions. **A** Stacked bar plot of the ancestral relationships among 341 accessions (K = 2). **B** Delta K plotted against putative K ranging from 2 to 9. **C** Genetic diversity visualization in association panel exhibited by principal component analysis. PC1, principal component 1; PC2, principal component 2; PC3, principal component 3

As calculated by the STRUCTURE software, the peak of the broken line graph was observed at k = 2 (Fig. 1A, B), suggesting that the association panel can be divided into two subgroups. Principal component analysis (PCA) plots of the first three components clearly differentiate wheat accessions into two sub-populations, which align with the population structure results (Fig. 1C). The markers were distributed across the entire genome after filtering out low-quality ones (Fig. S2A). The LD was estimated to be approximately 4 Mb for the entire genome when the LD decay distance reached r^2^ = 0.5 (Fig. S2B), which was the support interval used to declare significant SNPs associated with a given target trait.

### Genome-wide associations

**Fig. 2:**
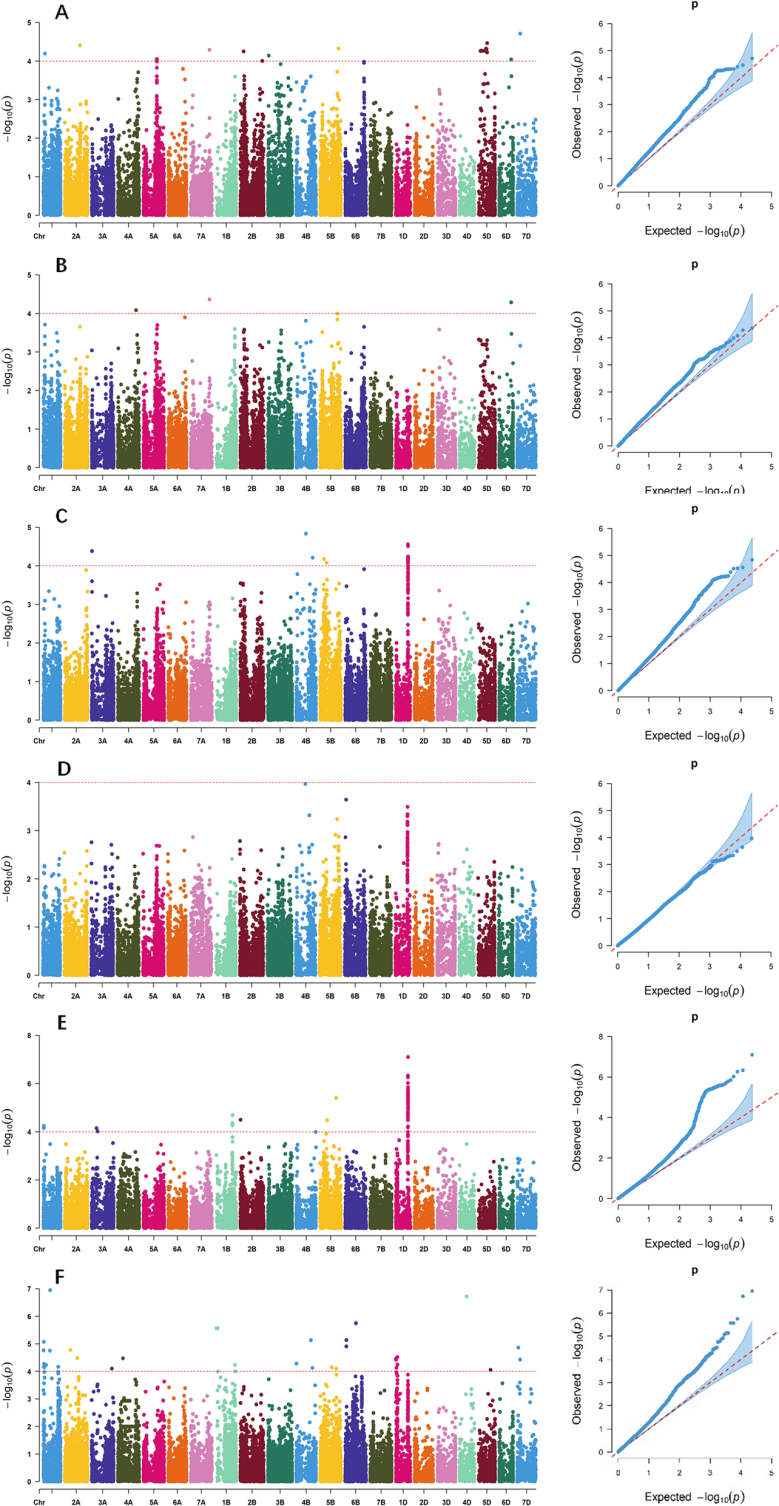
Manhattan and quantile-quantile (Q-Q) plots for protein quality traits identified through genome-wide association analysis using BLUP values. A horizontal line represents the significance threshold at which markers were considered associated with a trait (*P* < 1E-4, = 4). **A**, **B**, **C**, **D**, **E**, **F** Manhattan, and Q-Q plots for grain protein content, flour protein content, wet gluten content, dry gluten content, gluten index, and Zeleny sedimentation value, respectively

After filtering out the low-quality SNP markers, a total of 23,143 SNPs were selected for GWAS. GWAS with a mixed linear model (MLM) revealed 143 marker-trait associations (MTAs) for all protein quality traits except for dry gluten content based on BLUP values (Fig. 2, Table S7). These MTAs were distributed across all chromosomes except for 6 A, 2D, and 3D, and explained the phenotypic variation of 4.73 − 10.73% (Table S7). GWAS was also conducted for each environment (Tables S7, S12). A total of 97 association signals were consistently identified in two or more individual environments, which were considered stable. On the basis of the whole genomic LD decay distance (approximately 4 Mb) of the 341 accessions, significant SNPs within a 4 Mb region were combined into a single QTL. Thus, 43 loci were identified for the five quality traits (Table 3). These loci include 4 for grain protein content, 2 for flour protein content, 1 for wet gluten content, 4 for gluten index, and 32 for Zeleny sedimentation value. The *6D_410156938* marker has revealed a significant and stable MTA for both grain protein content and flour protein content. Similarly, the *1D_412227592* and *1D_415401424* markers have shown significant and stable MTAs for both wet gluten content and gluten index. The loci where these markers were located were considered pleiotropic QTL (Table 3).

**Table 3:** Significant single-nucleotide polymorphisms (SNPs) for protein quality traits were identified through genome-wide association analysis in multiple environments

Trait	Multi-environment	Marker	*P*-value					*R*^*2*^(%)				
			E1	E2	E3	E4	E5	E1	E2	E3	E4	E5
Grain protein content	E2 E5	*6D_410156938*		9.41E-05			9.05E-05		6.00			5.64
	E3 E5	*2A_517926919*			4.31E-05		3.94E-05			6.00		6.28
		*3B_27229830*			1.29E-05		7.25E-05			7.02		5.78
		*5A_455461352*			3.54E-05		9.12E-05			6.37		5.64
Flour protein content	E1 E5	*7A_647143478*	8.77E-05				4.34E-05	5.70				6.10
	E2 E5	*6D_410156938*		4.40E-05			5.15E-05		6.10			6.00
Wet gluten content	E2 E5	*1D_412227592*		8.46E-05			2.78E-05		5.68			6.39
		*1D_415401424*		3.65E-05			3.11E-05		6.21			6.32
Gluten index	E1 E2 E3 E5	*1D_411727551*	7.57E-05	2.25E-05	9.99E-06		2.87E-06	5.79	6.56	7.30		7.82
		*1D_412023762*	5.85E-05	6.24E-05	9.15E-06		2.3E-06	5.95	5.92	7.36		7.96
		*1D_415704212*	1.43E-05	1.08E-05	2.36E-05		3.54E-06	6.84	7.02	6.74		7.69
		*1D_415766966*	4.12E-05	5.02E-07	2.1E-05		8E-08	6.58	9.34	7.56		10.73
	E2 E3 E5	*1D_411237031*		1.95E-05	6.96E-05		6.51E-06		6.65	6.03		7.30
		*1D_411404496*		1.95E-05	3.78E-05		2.19E-05		6.70	6.48		6.66
		*1D_412031073*		1.19E-05	8.5E-05		1.65E-06		6.96	5.90		8.17
		*1D_412092560*		1E-05	1.51E-05		4.53E-06		7.07	7.03		7.53
		*1D_412136490*		8.3E-06	4.46E-05		2.58E-06		7.23	6.37		7.95
		*1D_412160361*		4.19E-06	4.09E-05		5.1E-06		7.63	6.38		7.46
		*1D_412178540*		2.62E-05	5.34E-05		2.28E-05		6.46	6.21		6.51
		*1D_412181832*		7.98E-05	6.98E-05		1.51E-05		5.80	6.09		6.78
		*1D_412227592*		7.28E-07	2.73E-05		5.44E-07		8.75	6.64		8.89
		*1D_413224542*		4.52E-07	3.32E-05		4.69E-07		9.06	6.52		8.98
		*1D_413292355*		3.63E-06	6.04E-05		3.79E-06		7.72	6.13		7.64
		*1D_413311188*		2.51E-05	3.62E-05		4.05E-06		6.49	6.46		7.60
		*1D_413729079*		3.63E-06	5.87E-05		2.62E-06		7.72	6.14		7.88
		*1D_413934525*		2.09E-05	1.81E-05		9.34E-06		6.61	6.91		7.07
		*1D_414144652*		1.58E-05	6.27E-05		2.43E-06		6.78	6.10		7.93
		*1D_414286643*		1.17E-05	4.99E-05		4.15E-06		6.99	6.25		7.59
		*1D_414451522*		2.43E-05	3.64E-05		9.46E-06		6.51	6.46		7.07
		*1D_414500837*		4.17E-06	5.99E-05		1.42E-06		7.63	6.13		8.27
		*1D_414573947*		4.27E-06	5.45E-05		4.87E-06		7.62	6.19		7.49
		*1D_414708956*		4.28E-06	5.92E-05		3.51E-06		7.61	6.14		7.69
		*1D_415126157*		5.2E-06	3.47E-05		4.06E-06		7.49	6.49		7.60
		*1D_415401424*		2.37E-06	3.96E-05		2.66E-06		7.99	6.40		7.87
		*1D_415459431*		2.01E-05	1.68E-05		7.36E-06		6.63	6.96		7.22
		*1D_415749624*		2.19E-05	2.29E-05		4.66E-06		6.58	6.76		7.51
		*1D_416033546*		4.26E-06	6.01E-05		5.19E-06		7.62	6.13		7.44
		*1D_416093683*		4.58E-06	3.44E-05		3.21E-06		7.57	6.49		7.75
		*1D_416101167*		5.41E-05	3.32E-06		1.04E-05		6.01	8.03		7.00
		*1D_416212526*		2.76E-05	3.71E-05		1.22E-05		6.43	6.44		6.90
	E1 E3 E5	*1D_411320325*	1.68E-05		9.41E-05		9.62E-07	6.76		5.91		8.56
	E1 E2 E5	*1D_411192168*	9.68E-05	2.83E-05			5.8E-06	5.64	6.41			7.38
		*1D_415213648*	6.5E-05	1.12E-05			3.24E-06	5.99	7.15			7.90
	E1 E5	*1B_559539573*	3.57E-05				2.06E-05	6.39				6.79
	E2 E5	*1A_20940731*		6.18E-05			0.00007		5.92			5.81
		*1B_561268092*		6.1E-05			4.41E-05		4.89			5.05
		*1D_411295558*		5.86E-05			3.19E-06		5.95			7.75
		*1D_411366902*		8.32E-05			3.27E-05		5.73			6.29
		*1D_411374953*		2.17E-05			1.67E-05		6.58			6.71
		*1D_412072331*		6.11E-06			4.49E-06		7.39			7.54
		*1D_412196822*		1.43E-05			2.67E-05		6.87			6.43
		*1D_412288344*		1.51E-05			1.83E-05		6.81			6.65
		*1D_412495297*		1.37E-05			0.00001		6.96			7.18
		*1D_414493743*		7.55E-06			1.93E-06		7.25			8.08
		*1D_415059649*		1.34E-05			1.82E-05		6.89			6.65
		*1D_415158238*		5.11E-05			0.00006		6.04			5.90
		*1D_415290059*		7.29E-06			7.47E-06		7.27			7.21
		*1D_415298532*		2.31E-06			2.9E-06		8.01			7.82
		*1D_415466562*		1.1E-05			9.83E-06		7.01			7.04
		*1D_415586977*		6E-06			4.19E-06		7.40			7.58
		*1D_415645580*		4.56E-06			3.86E-06		7.57			7.63
		*1D_416081310*		7.17E-06			6.16E-06		7.28			7.34
Zeleny sedimentation value	E1 E2 E3 E4 E5	*4D_238181951*	5.66E-07	1.65E-05	7.22E-06	1.2E-07	1.91E-07	7.72	5.67	6.41	8.69	8.34
		*6B_370150478*	3.16E-06	3.91E-05	8.7E-05	1.5E-07	1.77E-06	6.67	5.15	4.87	8.56	6.98
	E1 E2 E4 E5	*1A_225520951*	8.86E-05	5.75E-05		3.7E-06	1.78E-05	5.69	5.96		7.73	6.66
	E1 E3 E4 E5	*1A_9574254*	2.72E-05		1.46E-05	3E-06	1.73E-05	5.37		5.97	6.72	5.61
		*1A_21873794*	4.41E-06		7.54E-05	3.8E-05	8.45E-06	6.46		4.96	5.20	6.03
		*1A_236720351*	7.84E-09		7.17E-06	3E-07	1.13E-07	10.41		6.42	8.14	8.67
		*1B_53066005*	2.69E-06		3.06E-05	1.1E-06	2.71E-06	7.91		6.62	8.51	7.86
		*1D_232760*	3.8E-05		6.07E-05	9.7E-06	3.48E-05	5.18		5.11	6.01	5.20
		*1D_6299684*	2.33E-05		9.61E-05	3.4E-05	3.78E-05	5.46		4.81	5.26	5.14
		*4B_500743139*	1.54E-06		1.3E-05	4.6E-05	7.43E-06	7.10		6.04	5.07	6.11
	E1 E4 E5	*1A_33855698*	8.59E-05			9.1E-05	7.21E-05	4.69			4.67	4.76
		*1B_9992236*	4.66E-06			5E-07	2.71E-06	7.56			9.02	7.86
		*1D_49579197*	4.55E-05			8.2E-05	3.06E-05	6.11			5.76	6.32
		*2A_425759128*	2.12E-05			7.2E-06	3.25E-05	5.52			6.19	5.23
		*4B_550381492*	3.92E-05			6.9E-06	7.44E-05	5.15			6.22	4.75
		*6B_43515830*	6.46E-05			4.2E-05	7.36E-06	5.89			6.18	7.22
		*7D_37949527*	6.82E-06			8.2E-06	1.37E-05	6.20			6.11	5.74
	E1 E3	*1A_41516924*	1.34E-05		5.97E-05			5.79		5.10		
		*1B_67642598*	1.16E-05		8.64E-05			7.18		6.09		
	E1 E5	*1A_96454293*	2.64E-05				5.86E-05	5.39				4.89
		*1D_56039301*	9.44E-05				5.62E-05	5.65				5.94
		*2A_192541883*	4.68E-05				1.66E-05	6.10				6.71
		*3A_686822185*	4.36E-05				7.93E-05	5.10				4.73
		*4A_177632748*	9.35E-06				3.34E-05	6.01				5.22
		*5B_400532969*	3.69E-05				7.16E-05	6.25				5.80
		*5D_408811401*	3.1E-06				8.82E-05	7.82				5.66
	E4 E5	*1A_12815998*				2.5E-06	6.74E-05				7.99	5.92
		*1A_31178918*				5.3E-05	0.000053				4.99	4.94
		*1A_508723612*				4.5E-05	6.86E-05				6.14	5.82
		*1D_19807137*				2.5E-05	7.45E-05				6.50	5.77
		*7D_96433791*				4.9E-07	3.75E-05				9.88	6.87
	E1 E4	*1A_34406785*	3.95E-05			5.8E-05		5.15			4.93	
		*1A_291896187*	6.13E-05			4.3E-05		5.93			6.19	
		*3A_65406649*	7.08E-05			8.5E-05		4.80			4.71	
	E3 E4	*3B_28179353*			3.36E-05	8.9E-05				5.45	4.69	

E1, 2020_EM; E2, 2020_QT; E3, 2021_EM; E4, 2021_QT, which represent the cropping seasons in E’min (EM) and Qitai (QT) for the years 2019–2020 and 2020–2021, respectively; E5, BLUP, the best linear unbiased predictor of protein quality traits in 341 wheat accessions during two cropping seasons across two environments

### Haplotype analysis

**Fig. 3:**
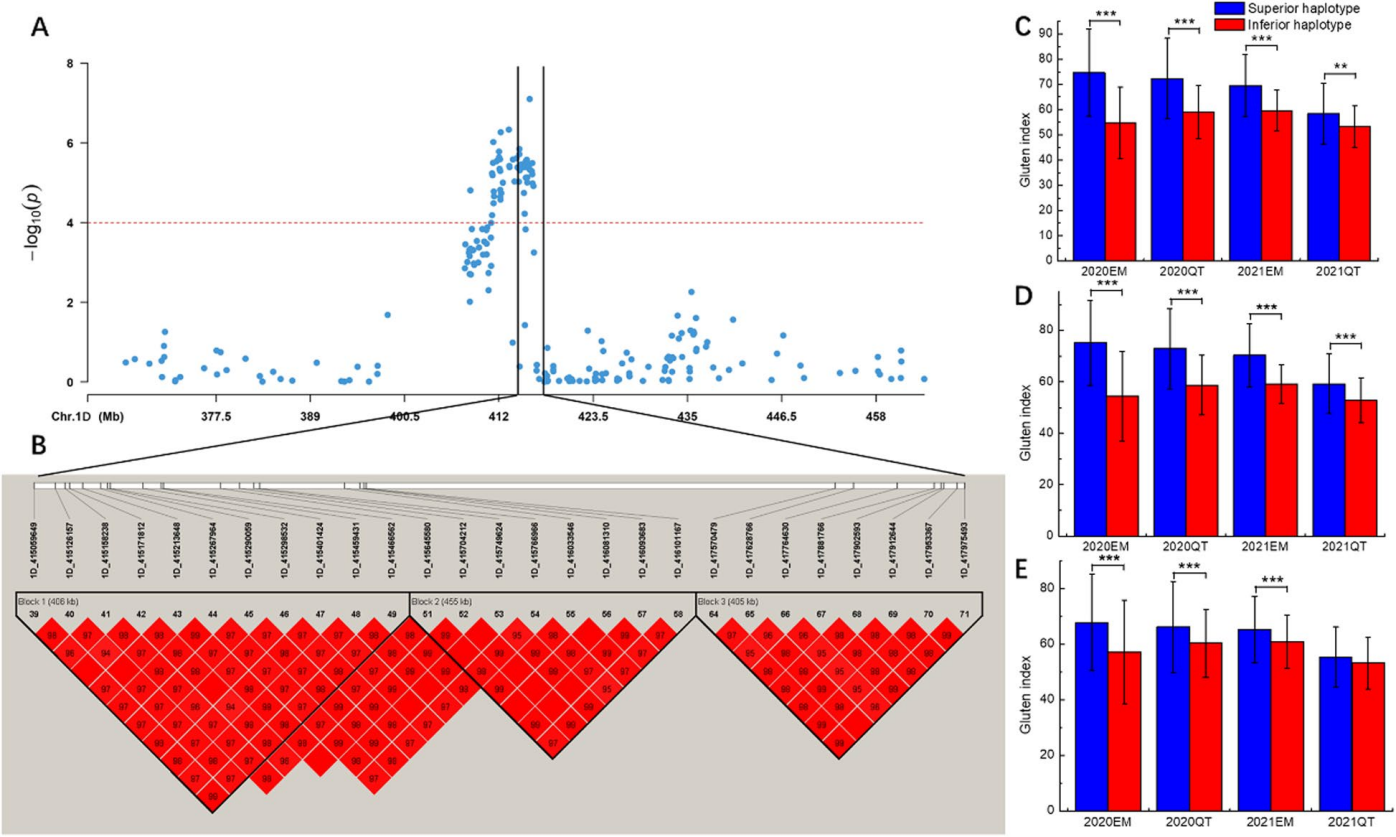
Haplotype analysis. **A** Local Manhattan plots. **B** in haplotype analysis of multi-environment significant SNPs associated with the gluten index. The color represents the linkage between SNPs, and the deeper color means the higher linkage between SNPs. **C**, **D**, **E** phenotypic effects of haplotypes in blocks 1, 2, and 3, respectively, across different environments. *** indicates significant differences *P *< 0.001; ** indicates significant differences *P* < 0.01. 2020_EM, 2020_QT, 2021_EM, and 2021_QT, represent 2019–2020 and 2020–2021 cropping seasons in E’min (EM), and Qitai (QT), respectively

Stable SNPs were selected to group the accessions based on their genotypes, and a *t*-test was used to determine the significance of genotypic effects on the traits (Table S8). SNPs identified for the gluten index within the 412–416 Mb interval on chromosome 1D revealed a significant difference (*P* < 0.001) in traits between the accessions harboring different alleles in all environments (Fig. 3A; Table S8). Haplotype analysis conducted in this genetic region identified three blocks (Fig. 3B; Table S9). There are 11, 8, and 8 SNPs clustered in blocks 1, 2, and 3, respectively. The accessions were categorized according to their haplotypes, and *t*-tests showed that accessions with a superior haplotype have a significantly higher gluten index than those with inferior haplotypes (*P* < 0.001) in all three blocks across environments, except for 2021_QT in block 3 (Fig. 3C, D, E).

## Validation of candidate SNP

**Fig. 4:**
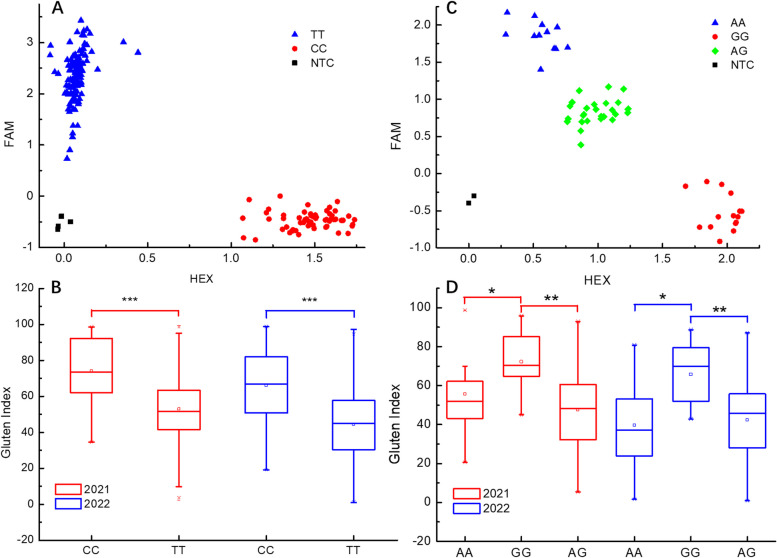
Kompetitive allele-specific PCR (KASP) verification of a significant single nucleotide polymorphism (SNP) associated with the gluten index. **A** Scatter plot of KASP marker *1D_415401424*. **B** The variance of the gluten index for accessions with different alleles (*1D_415401424*). **C** Scatter plots of KASP marker *1D_415704212*. **D** The variance of the gluten index for accessions with different alleles (*1D_415704212*). Red dots and blue triangles represent the homozygous genotypes; green diamonds represent heterozygous genotypes; black squares in the bottom left of the plot indicate the no-template control. *** indicates significant differences at *P *< 0.001; ** indicates significant differences at *P* < 0.01; * indicates significant differences at *P* < 0.05

KASP markers were designed for the significant association stable SNPs and were genotyped on a randomly selected subset from a validation panel. The SNP markers *1D_415401424* and *1D_415704212* were identified in the GWAS as significantly associated with the gluten index (Table 3). Furthermore, these two markers were found to be located in blocks 1 and 2, respectively, in haplotype analyses (Fig. 3, Table S9). The KASP marker for *1D_415401424* clearly clusters the accessions based on their alleles. Accessions with the CC allele showed significantly higher gluten index than those with the TT allele in both 2021 and 2022 (Fig. 4A, B). KASP marker for *1D_415704212* successfully genotyped the accessions into AA, GG, and AG categories, revealing highly significant phenotypic differences among accessions with different alleles (Fig. 4C, D). Additionally, the KASP marker developed for other SNPs significantly associated with the gluten index (Fig. S3) and Zeleny sedimentation value (Fig. S4) also successfully genotyped the accessions, with distinct allelic genotypes exhibited significant phenotypic differences.

### Candidate genes identification

**Fig. 5:**
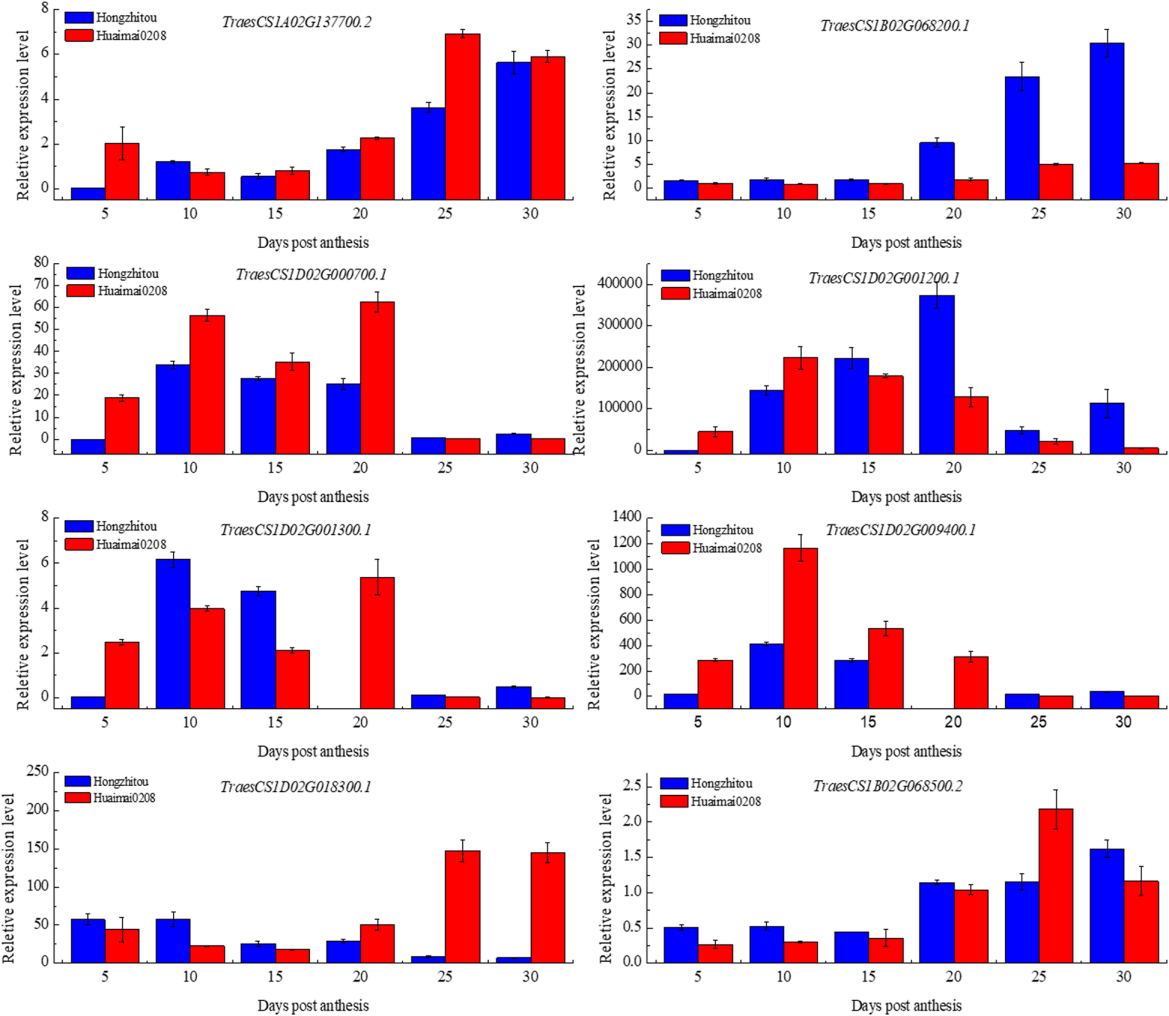
Expression of candidate genes in seeds at 5, 10, 15, 20, 25, and 30 days post-anthesis of accessions with different protein quality

A total of 621 genes were searched in the 4 Mb sequences flanking the stable SNPs associated with five protein quality traits. Among them, the genes located both before and after the SNP, as well as those that overlap with the SNP, are shown in Table S10. Utilizing RNA-seq data from public expression databases and their functional annotations, along with considering genes that overlap with significant SNPs, 13 candidate genes were selected for qPCR validation (Fig. S5; Table S11). Eight of them were found to be differentially expressed in the seeds post-anthesis of two distinct protein quality materials (Fig. 5).

## Discussion

### Phenotype variation and population structure

Grain protein quality is a complex trait that varies among genotypes and is strongly influenced by the environment [6]. In this study, all protein quality traits exhibited significant differences among genotypes, locations, and years (Table S6). Considerable CV was observed for the gluten index, indicating significant phenotypic variation among the genotypes, particularly in the 2020EM and 2020QT environments. This suggests that the genetic variation of the gluten index within the associated population is substantial and is also strongly affected by environmental factors, as evidenced by a lower heritability of 0.67 for the gluten index. Heritability estimates for protein quality traits in our study were high (Table 1), which agrees with the results of previous reports [40], indicating that these traits are highly heritable [41], making them easier to improve through selection in breeding program. There were significant correlations between different traits (Table 2), which were consistent with those found in other recent studies [37, 42–44].

Population stratification and familial relatedness pose significant challenges in controlling false positives in GWAS [44, 45]. In the present study, both STRUCTURE analysis and principal component analysis yielded consistent results regarding population structure, with the 341 genotypes divided into two subgroups (Fig. 1). The LD within a population forms the basis of GWAS and may vary across different populations. This variation is influenced by several factors, including population size, genetic drift, admixture, selection, mutation, non-random mating, pollination behavior, and recombination frequency [45]. In this study, LD decay was estimated to be approximately 4 Mb for the entire genome (Fig. S2B), which is larger than that observed in other plant species, such as maize and rice [44]. This may be attributed to the fact that wheat is a self-pollinating species with a considerably large genome [44].

### Association with protein content

The grain protein content plays a paramount importance in both nutritional value and end-use quality [46]. Both the grain protein content and flour protein content were genetically dissected in this study. GWAS identified four stable loci associated with grain protein content located on chromosomes 6D (410 Mb), 2 A (517 Mb), 3B (27 Mb), and 5 A (455 Mb), and two loci for flour protein content on chromosomes 7 A (647 Mb) and 6D (410 Mb) (Table 3). Highly significant correlations among certain pairs of traits were consistent with the clustering of QTL for those traits in specific genomic regions [40]. In this study, we found a significant correlation between grain protein content and flour protein content, with both traits sharing QTL locations on chromosome 6D. The results of GWAS in our study were consistent with previous research [11, 42]. For example, Hao et al. [44] identified QTLs for protein content located at 479.28–479.32 Mb on chromosome 5 A through genome-wide association mapping involving 253 samples. This location is about 24 Mb away from one of the loci (*5A_455461352*) identified in the present study (Table 3). Kartseva et al. [10] also identified QTLs associated with protein content on chromosomes 5 A (488 Mb) and 7 A (694 Mb) through GWAS analysis in a panel of 255 wheat accessions from 27 countries. In addition, Nigro et al. [11] identified stable QTL for protein content on chromosomes 3B and 5 A in durum wheat germplasm. Previous studies have reported genetic loci associated with protein content on all wheat chromosomes [10, 34, 40, 47–50]. However, the limited number of QTLs for protein content observed in this study may be attributed to the low genetic diversity among the genotypes, possibly due to the fixation of high-protein-content QTL alleles (Table 1).

### Association with gluten content

The majority of the protein in wheat grain is gluten protein, accounting for approximately 80% of total protein. The gluten protein consists of a network of high- and low-molecular-weight (HMW and LMW) glutenins and monomeric gliadins [51]. The HMW glutenins, Glu-A1, Glu-B1, and Glu-D1 are located on the long arms of chromosomes 1 A, 1B, and 1D, respectively. On the other hand, the LMW glutenins, Glu-A3, Glu-B3, and Glu-D3 are located on the short arms of chromosomes 1 A, 1B, and 1D, respectively [39, 48]. Wheat prolamins are encoded by several loci on group one and six chromosomes [52]. QTLs for gluten content have also been identified on chromosomes 2 A, 2B, 2D, 3 A, 4B, 5 A, 5D, 6 A, and 7 A [37, 44, 48, 49]. In this study, only one locus associated with wet gluten was identified on chromosome 1D. However, no loci were identified for dry gluten content (Table 3). This may be due to a low frequency of allelic variants in our association panel, which reduces the power of GWAS to identify an association between an SNP and a trait [39, 53].

### Association with gluten index

The gluten index value reflects the quality of glutenin and gliadin. High-quality glutenin provides the tensile resistance of the dough, while high-quality gliadin provides the adhesion required for dough fluidity and ductility. Four loci associated with the gluten index have been identified on chromosomes 1 A, 1B, and 1D (2) (Table 3). Consistent with Deng et al. [54], who identified five QTLs for the gluten index mapped on chromosomes 1 A, 1B, 1D, and 6 A, explaining 3.96–22.44% of the phenotypic variance using NG lines derived from Gaocheng 8901 and Nuomai 1. Previous studies have also identified gluten index QTLs on chromosomes 2D, 3 A, 3B, 4 A, 4B, 6 A, and 7B, which explained high phenotypic variation [48, 55]. However, these QTLs were not detected in our research. In the present study, a total of 51 association SNPs for gluten index were identified from 411 Mb to 418 Mb on chromosome 1D (Table 3). These alleles are inherited together, and the opportunity for contemporary recombination is minimal [56]. Haplotype analysis conducted in this genetic region identified three distinct blocks. Accessions with superior haplotypes showed a significantly higher gluten index compared to those with inferior haplotypes across all environments, except for the 2021_QT in block 2 (Fig. 3). This indicates that the combination of GWAS and haplotype analysis is more effective than single-marker-based analysis in estimating statistical significance (better p-values) and allelic effects [57].

### Association with Zeleny sedimentation value

The gluten content and quality can be estimated using the Zeleny sedimentation value [39]. Large sedimentation volumes indicate high gluten content and strength [12]. In our study, a total of 32 QTLs were identified for the Zeleny sedimentation value on chromosomes 1 A, 1B, 1D, 2 A, 3 A, 3B, 4 A, 4B, 5B, 5D, 6B, and 7D. Most of them are located on the homoeologous chromosome group one (i.e., 1 A, 1B, and 1D) (Table 3). These findings are consistent with previous research [38, 39] and may be linked to the major storage proteins of glutenin subunits (Glu-1 and Glu-3) and γ- and ω-gliadins (Gli-1 and Gli-3) loci, which are distributed on the short and long arms of homoeologous chromosome group one [58]. SNP marker *1A_508723612*, identified in the present study (Table 3), is located just 1,372 bp away from *TraesCS1A01G466500LC.1*, which encodes the protein Glu1Ay [43]. QTLs for Zeleny sedimentation value have also been reported on chromosomes 2 A, 2B, 3D, 4 A, 4D, 6B, and 7 A [39, 42, 59, 60]. In our study, we also identified QTLs for Zeleny sedimentation value on these chromosomes, but they were located in different genetic regions. Significant association loci on chromosomes 2D, 3 A, 5 A, 7B, and 7D were not previously reported in durum wheat and may represent novel findings for Zeleny sedimentation value.

### KASP marker development

KASP can be used for genotyping a wide range of species for various purposes, including quality control analysis, quantitative trait loci mapping, marker-assisted recurrent selection, and allele mining [20]. KASP markers have obvious advantages over other DNA molecular markers. Previous studies have shown that KASP assays are 45 times faster than gel-based PCR markers [61]. For the development of markers to be used for high-throughput genotyping, the SNPs significantly associated with protein quality traits identified in the GWAS were selected, and KASP assays were designed accordingly. All KASP assays were validated against phenotype data. When accessions with different alleles were clearly separated and exhibited significant differences in the phenotypic effects of both alleles within the validation panel, the KASP assay was deemed successful. There were 11 SNPs successfully converted to KASP markers (Fig. 4; Figs. S3, S4). This will significantly accelerate the selection and pyramid of favorable alleles in wheat breeding programs.

### Candidate genes

Many QTLs associated with wheat quality traits have been identified; however, only a few putative candidate genes have been pinpointed within these genomic regions [44]. Different gene expression patterns exist for the seed coat, endosperm, and embryo during the grain-filling process [62]. In this study, eight candidate genes were identified based on their differential expression in whole seeds after anthesis (Fig. 5). Previous study has reported that the accumulation changes in grain protein content during wheat grain filling follow a trend “high-low-high” [63]. The study conducted by Guo et al. [34] found that this trend is less influenced by environmental factors and genetic background. In our study, candidate genes were differentially expressed either at 5 and 10 days post-anthesis or at 20 and 25 days post-anthesis, which is consistent with the period of rapid protein accumulation.

Previous studies have shown that protein accumulation during grain development is influenced by regulation at both the transcriptional and post-transcriptional levels, which involves various transcription factors (TFs) regulating prolamin genes, such as MYB TFs, AP2, and NAC TFs, and some important protein modifications, such as N-glycosylation, folding, assembly of proteins, and protein bodies [64–66]. In our study, candidate genes for protein quality traits were screened based on differential gene expression. Three candidate genes were coding genes for grain proteins, including gliadin/avenin-like seed protein, gamma gliadin-D2, and delta gliadin-D1. Tobamovirus multiplication protein 2 A (TOM2A) is encoded by *TraesCS1A02G137700.2*. A previous study has reported that *TOM2A* genes may play fundamental roles in plant development or in responses to stress [67]. The co-chaperone protein p23, encoded by *TraesCS1B02G068200.1*, is historically known as a co-chaperone of heat shock protein 90 (HSP90), exerts some of its critical functions in an HSP90-independent manner. It was also a previously unidentified transcription factor of *COX-2*, as reported by Yu et al. [68]. Rhodopsin is encoded by *TraesCS1B02G068500.2*, which is the pigment-containing sensory protein that converts light into an electrical signal. Rhodopsin is found in a wide range of organisms, from vertebrates to bacteria [69]. The V-type proton ATPase proteolipid subunit is encoded by *TraesCS1D02G018300.1*. Vacuolar-type ATPases (V-ATPases) serve as the primary proton pumps responsible for acidifying and maintaining the pH of intracellular compartments, which are important for fundamental cellular processes [70]. The roles of the aforementioned genes in protein synthesis deserve further research in the future.

## Conclusions

To identify genomic regions responsible for protein quality traits in winter wheat and to provide reliable alleles for marker-assisted selection (MAS) in the improvement of wheat protein quality, GWAS was conducted on six protein quality traits using 341 wheat accessions and the wheat 40 K SNP array. A total of 97 significant and stable SNPs were identified at 43 loci for protein quality traits. The most significant associations were identified on chromosomes 1 A, 1B, and 1D. Six KASP markers for gluten index and five KASP markers for Zeleny sedimentation values were successfully developed. Additionally, 8 candidate genes that may be related to protein accumulation during grain development were screened at significantly associated loci. Our study will provide valuable insights for wheat breeding programs in China and globally.

## Methods

### Plant materials

An association panel consisting of 341 winter wheat genotypes, which includes a selection of cultivars and advanced breeding lines from various provinces of China, as well as two genotypes imported from Italy (Table S1). This association panel was selected from our existing germplasm resources based on a wide range of phenotypic diversity. The validation of KASP markers was conducted in 200 winter wheat materials composed of different wheat varieties/lines (referred to as the validation population). Among these, 193 genotypes are distinct from the associated population, while the remaining 7 genotypes are part of the associated population. The SNPs of these 7 genotypes at the KASP marker development site are known, so they can verify the accuracy of KASP marker genotyping. The validation panel was used to verify the success or failure of the KASP marker (Table S2).

### Phenotyping

The association panel was evaluated at two sites, including the Institutes of Agricultural Sciences in E’min and Qitai, Xinjiang, China, during the years 2019–2020 and 2020–2021 (hereafter referred to as 2020_EM, 2020_QT, 2021_EM, and 2021_QT, respectively). The validation accessions were planted in Shihezi, Xinjiang, China, during the years 2020–2021 and 2021–2022. Protein indicators were measured after harvest. The experiment utilized an alpha-lattice design with two replications. Each replication consisted of 18 incomplete blocks, with each block comprising 19 genotypes. Each genotype was grown in a plot measuring 1.8 m in length, arranged in 8 rows with 0.25 m of spacing between them, with each row sown with 40 seeds. Recommended management practices were applied to the trials at their respective locations. Plots were hand-harvested at maturity, and the grain was stored at 4 °C. Using the MLU202 Mill (Wuxi, China), the grain was ground and passed through a 0.1-micron sieve. The flour samples were stored in airtight containers.

The grain protein content and flour protein content were measured using the Foss Infratech TM 4100 Grain Analyzer.

Gluten characteristics of flour, including wet gluten content (WGC), dry gluten content, and gluten index, were measured using a gluten analysis instrument (Perten Instruments 2200, Huddinge, Sweden) in accordance with AACC Method 38 − 11 [42].

Zeleny sedimentation was conducted in accordance with AACC Method 56–63 [4].

### Genotyping and quality control

DNA was extracted from two-week-old seedlings for each accession. The associated accessions were genotyped using the Wheat 40 K breeding array by the Mol-Breeding Company in Tianjin, China (http://www.molbreeding.com/). The SNP markers with a minor allele frequency (MAF) of less than 5% and missing data of more than 10% were removed from the analysis.

### Population structure and linkage disequilibrium

Population structure was estimated using the Bayesian model-based clustering program STRUCTURE v2.3.4 [71]. We used 10 subgroups (K = 1–10) with five independent runs for each subgroup using a burn-in period of 10,000 iterations followed by 10,000 Monte Carlo iterations. The most likely number of subgroups was inferred based on an ad hoc statistic (DeltaK) [72], using STRUCTURE HARVESTER [73].

The PCA analysis and pairwise linkage disequilibrium (LD) were calculated using TASSEL 5.0 [74], with the whole-genome filtered genotypes shared by the accessions. The LD results from TASSEL were used to estimate and plot LD decay over distance (bp) in R, as described by Hill and Weir [75]. The LD decay distance in the whole genome was estimated by setting the *r*^2^ threshold at half of the LD decay [44, 45].

### GWAS

GWAS was performed using the mixed linear model (MLM) in TASSEL 5.0 [74]. The MLM controlled for both population structure (Q-matrix) and kinship matrix (K-matrix) to avoid false positives. The kinship coefficient between each pair of accessions was estimated in TASSEL 5.0 using the Loiselle algorithm [76]. Markers were defined as being significantly associated with the trait when the significance test reached *P* < 0.0001 (-log10(*P*) ≥ 4). If multiple SNPs controlling the trait of interest were identified within one LD block on a chromosome segment, they could all be referred to as QTL [77]. Single-locus contributing to multiple phenotypic traits was considered pleiotropic loci, while markers identified in at least two environments were considered stable loci, as reported in several studies [18, 78]. The Manhattan and Q-Q plots were constructed using the qqman package in R [79].

### Haplotype analysis

The haplotype analysis of significant SNP loci was performed using Haploview software version 4.2 [80]. The blocks were generated by Haploview based on confidence intervals, as described by Gabriel et al. [81].

### Validation of KASP markers

The SNP consistently identified in different environments was selected, and a KASP assay was designed. Two allele-specific forward primers and one common reverse primer were designed using the online software Polymarker (http://www.polymarker.info/). The standard FAM (5′ GAAGGTGACCAAGTTCATGCT 3′) and HEX (5′ GAAGGTCGGAGTCAACGGATT 3’) tails were added to the 5′ end of the two allele-specific primers, respectively. Before applying PCR, the primer assay mixture was prepared. It consisted of 46 µL of ddH_2_O, 30 µL of common primer (100µM), and 12 µL of each tailed primer (100µM). The final volume of the PCR reaction was 10.14 µL, containing 5 µL of diluted DNA (30 ng/µL), 5 µL of 2x KASP master mix, and 0.14 µL of KASP assay mix. PCR cycling was performed using the following protocol: hot start at 94 °C for 15 min, followed by ten touchdown cycles (94 °C for 20 s; touch down at 61 °C initially and decreasing by −0.6 °C per cycle for 60 s), followed by 30 additional cycles of annealing (94 °C for 20 s; 55 °C for 60 s) [82, 83].

SNP genotyping of KASP markers was performed in 96-well formats using an ABI 7500 instrument, following the touchdown thermal cycling protocol described by the manufacturer (LGC Genomics, Beverly, MA, USA). The genotypic data were analyzed using the ABI 7500 instrument, while also incorporating subjective assessment based on fluorescence values [84].

In the initial run of the 96-well plate, if the KASP marker can clearly distinguish the validation accessions and there are significant phenotypic differences among accessions with different alleles, the subsequent 96-well plate will be run consecutively. Otherwise, the validation process will conclude. This means that an ideal KASP marker will genotype 188 randomly selected accessions from the validation panel, while a less effective marker will only genotype 94 accessions. A *t*-test was applied to detect significant differences in traits between the allele types, based on the SNPs. The primer pair sequences for PCR amplification are listed in Table S3.

### Candidate genes identified

To identify potential candidate genes associated with protein quality traits, all genes within the LD region of stable SNPs (2 Mb upstream and downstream of the SNP positions, respectively) were searched through the IWGSC database (http://www.wheatgenome.org/). RNA-seq data from wheat aleurone and starchy layers at 6, 9, and 14 days post-anthesis [85] in the publicly available Expression Atlas database (https://www.ebi.ac.uk/gxa/experiments/E-GEOD-38344/Results) was used to analyze the gene expression profiles of these genes. Based on functional annotations, genes that were highly and differentially expressed at 6, 9, and 14 days post-anthesis, and genes that overlapped with the stable SNPs, were selected for further analysis. The expression levels of these selected genes were measured using qPCR in 5, 10, 15, 20, 25, and 30 days seeds of two different protein quality materials. These two cultivars were Hongzhitou and Huaimai 0208, which have high and low protein content, respectively (Table S4).

The functional annotation of genes was conducted using the UniProt Protein Database (https://www.uniprot.org/) and the Ensemble Plants Database (http://plants.ensembl.org/Triticum_aestivum/Gene).

Total RNA was isolated from leaves using the TRIzol reagent (Invitrogen, USA), following the manufacturer’s instructions. The concentration of total RNA was measured spectrophotometrically using a NanoDrop instrument (Thermo Scientific NanoDrop 2000 C Technologies, Wilmington, USA), and the purity was assessed using the A260/A280 and A260/A230 ratios provided by the NanoDrop. Reverse transcription was carried out using a PrimeScript™ First-Strand Complementary DNA (cDNA) Synthesis Kit (TaKaRa, Japan).

qRT-PCR was performed using the iCycler iQ™ Multicolor PCR Detection System (Bio-Rad, Hercules, CA, USA). qPCR was performed with cDNA in triplicate on 96-well plates using SYBR^®^ Premix Ex Taq™ II (TaKaRa). Each reaction (20µL) consisted of 10µL of SYBR^®^ Premix Ex Taq™ II, 1 µL of diluted cDNA, 0.4 µL of forward and reverse primers, and 8.2 µL of H_2_O. The qPCR cycling conditions were as follows: an initial denaturation at 95 °C for 2 min, followed by 40 cycles of denaturation at 95 °C for 5 s and annealing/extension at 57 °C for 32 s. Fluorescence data were collected during the 57 °C step. Actin (GenBank ID: LOC123114174) was used as a reference gene for wheat. The gene ID and primer sequences are provided in Table S5. Relative quantification of gene expression was calculated using the delta Ct method.

### Statistical analysis

A multi-environment trial analysis was conducted using R software to perform an analysis of variance (ANOVA).

The average of the trait was calculated using the Best Linear Unbiased Predictor (BLUP) method [86] with the R package lme4 [87].

Broad-sense heritability (*h*^*2*^) was estimated from variance components using the formula: *h*^*2*^ = σ^2^_G_ / (σ^2^_G_ + σ^2^_GE_/E + σ^2^_e_/rE), where σ^2^_G_, represents the genetic variance, σ^2^_GE_ represents the genotype-environment interaction variance, σ^2^_e_ represents the residual variance, E represents the number of environments, and r represents the number of replicates per line [88].

Pearson’s correlation between phenotypic traits was computed using SPSS version 22 (http://www.brothersoft.com/ibm-spss-statistics-469577.html).

Other ANOVA analyses and plots were conducted using SPSS version 22 and Origin version 8.0, respectively.

A gene expression heatmap was performed using TBtools [89].

## Supplementary Information

Supplementary Material 1.

## Data Availability

The data presented in the study are deposited in Figshare DOI: 10.6084/m9.figshare.24739221.
