# Supplementary material for: Genome-wide association analysis and KASP markers development for protein quality traits in winter wheat
Source: BMC Plant Biol. 2025 Feb 5;25:149. doi: 10.1186/s12870-025-06171-z (PMC11796262; doi:10.1186/s12870-025-06171-z)
Supplement: Supplementary file 1 — Supplementary Material 1. [file 12870_2025_6171_MOESM1_ESM.docx]

Table S1 The names of 341 accessions in the association panel

| Code | Name | Origination | Code | Name | Origination | Code | Name | Origination |
| --- | --- | --- | --- | --- | --- | --- | --- | --- |
| 1 | Xindong 18 | Xinjiang | 115 | 2013(86)/2-3-1 | Xinjiang | 229 | YY-103/2 | Xinjiang |
| 2 | Xindong 2 | Xinjiang | 116 | 2013(86)/7-1-1 | Xinjiang | 230 | YY-120/2 | Xinjiang |
| 3 | Xindong 14 | Xinjiang | 117 | 2013(94)/3-2-1 | Xinjiang | 231 | YY-121/2 | Xinjiang |
| 4 | Xindong 15 | Xinjiang | 118 | 2013(94)/3-2-2 | Xinjiang | 232 | YY-122/3 | Xinjiang |
| 5 | Xindong 16 | Xinjiang | 119 | 2013(98)/4-4-2 | Xinjiang | 233 | YY-123/2 | Xinjiang |
| 6 | Xindong 17 | Xinjiang | 120 | 2013(104)/1-1-2 | Xinjiang | 234 | YY-125/1 | Xinjiang |
| 7 | Xindong 18 | Xinjiang | 121 | 2013(104)/1-2-1 | Xinjiang | 235 | YY-126/1 | Xinjiang |
| 8 | Xindong 19 | Xinjiang | 122 | 2013(108)/1-1-1 | Xinjiang | 236 | YY-138/1 | Xinjiang |
| 9 | Xindong 20 | Hebei | 123 | 2013(110)/2-6-2 | Xinjiang | 237 | YY-139/3 | Xinjiang |
| 10 | Xindong 21 | Xinjiang | 124 | 2013(127)/2-1-1 | Xinjiang | 238 | YY-142/1 | Xinjiang |
| 11 | Xindong 22 | Xinjiang | 125 | 2013(130)/2-1-3 | Xinjiang | 239 | YY-234/2 | Xinjiang |
| 12 | Xindong 23 | Xinjiang | 126 | 2013(130)/2-3-2 | Xinjiang | 240 | M-3/4 | Xinjiang |
| 13 | Xindong 24 | Xinjiang | 127 | 2013(130)/2-4-1 | Xinjiang | 241 | Shidong 03112 | Xinjiang |
| 14 | Xindong 26 | Xinjiang | 128 | 2013(133)/3-2-2 | Xinjiang | 242 | Shidong 0358 | Xinjiang |
| 15 | Xindong 27 | Xinjiang | 129 | 2013(143)/1-3-2 | Xinjiang | 243 | Shidong 0821 | Xinjiang |
| 16 | Xindong 28 | Xinjiang | 130 | 2013(148)/2-3-1 | Xinjiang | 244 | Shidong 04104 | Xinjiang |
| 17 | Xindong 29 | Xinjiang | 131 | 2013(148)/5-2-2 | Xinjiang | 245 | Shidong 01162 | Xinjiang |
| 18 | Xindong 30 | Xinjiang | 132 | 2013(154)/1-1-2 | Xinjiang | 246 | Shidong 0349 | Xinjiang |
| 19 | Xindong 31 | Xinjiang | 133 | 2013(154)/4-1-1 | Xinjiang | 247 | Shidong 0451 | Xinjiang |
| 20 | Xindong 32 | Xinjiang | 134 | 2013(158)/1-2-1 | Xinjiang | 248 | Shidong 03194 | Xinjiang |
| 21 | Xindong 33 | Xinjiang | 135 | 2013(178)/1-4-1 | Xinjiang | 249 | Shidong 06510 | Xinjiang |
| 22 | Xindong 36 | Xinjiang | 136 | 2013(178)/8-3-1 | Xinjiang | 250 | JSH15-08 | Xinjiang |
| 23 | Xindong 37 | Beijing | 137 | 2013(183)/3-1-1 | Xinjiang | 251 | JSH606 | Xinjiang |
| 24 | Xindong 38 | Xinjiang | 138 | 2013(190)/6-2-1 | Xinjiang | 252 | JSH15-06 | Xinjiang |
| 25 | Xindong 40 | Xinjiang | 139 | 2013(202)/3-1-1 | Xinjiang | 253 | Xingmu1602 | Xinjiang |
| 26 | Xindong 41 | Xinjiang | 140 | 2013(205)/5-1-1 | Xinjiang | 254 | Xingmu 1603 | Xinjiang |
| 27 | Xindong 42 | Xinjiang | 141 | 2013(206)/2-1-1 | Xinjiang | 255 | Fengyuandong 3 | Xinjiang |
| 28 | Xindong 44 | Xinjiang | 142 | 2013(223)/3-2-2 | Xinjiang | 256 | 0858 | Xinjiang |
| 29 | Xindong 45 | Xinjiang | 143 | 2013(230)/6-4-2 | Xinjiang | 257 | SDWW-7 | Xinjiang |
| 30 | Xindong 48 | Xinjiang | 144 | 2013(230)/8-2-1 | Xinjiang | 258 | 03--40 | Xinjiang |
| 31 | Xindong 49 | Xinjiang | 145 | 2013(230)/10-1-3 | Xinjiang | 259 | 08/16 | Xinjiang |
| 32 | Xindong 51 | Xinjiang | 146 | 2013(230)/11-2-1 | Xinjiang | 260 | 04--1 | Xinjiang |
| 33 | Xindong 52 | Xinjiang | 147 | 2013(247)/5-1-1 | Xinjiang | 261 | Xinniang 14-6151 | Xinjiang |
| 34 | Xindong 53 | Xinjiang | 148 | 2013(247)/5-4-2 | Xinjiang | 262 | Xinniang 15-2217 | Xinjiang |
| 35 | Xindong 54 | Xinjiang | 149 | 2013(251)/1-1-1 | Xinjiang | 263 | Xinniang 606 | Xinjiang |
| 36 | Xindong 55 | Xinjiang | 150 | 2013(251)/5-3-1 | Xinjiang | 264 | 2015J/176 | Xinjiang |
| 37 | Xindong 57 | Xinjiang | 151 | 2013(255)/2-4-1 | Xinjiang | 265 | HWX1109 | Xinjiang |
| 38 | Xindong 60 | Xinjiang | 152 | 2013(255)/6-1-1 | Xinjiang | 266 | 09BC-3 | Xinjiang |
| 39 | Shidong 7 | Xinjiang | 153 | 2013(255)/8-4-2 | Xinjiang | 267 | Zhonglumai 666 | Shandong |
| 40 | Shidong 8 | Xinjiang | 154 | 2013(255)/10-1-2 | Xinjiang | 268 | JFM-2 | Xinjiang |
| 41 | Shidong 9 | Xinjiang | 155 | 2013(255)/11-2-1 | Xinjiang | 269 | Xm1607 | Xinjiang |
| 42 | Yinong 16 | Xinjiang | 156 | 2013(255)/11-2-3 | Xinjiang | 270 | CA15026 | Beijing |
| **43** | **Yinong 18** | Xinjiang | 157 | 2013(255)/11-2-5 | Xinjiang | 271 | BH5335 | Beijing |
| 44 | Kuidong 4 | Xinjiang | 158 | 2013(255)/11-2-7 | Xinjiang | 272 | 0868 | Xinjiang |
| 45 | Kuihua 1 | Xinjiang | 159 | 2013(255)/11-3-2 | Xinjiang | 273 | 0818 | Xinjiang |
| 46 | 95(20)/8-1-2 | Xinjiang | 160 | 2013(255)/11-8-2 | Xinjiang | 274 | Xinniang 152148 | Xinjiang |
| 47 | 99(55)/3-1-1 | Xinjiang | 161 | 2013(255)/11-9-2 | Xinjiang | 275 | Xinniang 152183 | Xinjiang |
| 48 | 99(79)/2-1-2 | Xinjiang | 162 | 2013(255)/12-2-8 | Xinjiang | 276 | JHD06 | Xinjiang |
| 49 | 2000(125)/4-2-1 | Xinjiang | 163 | 2013(255)/12-5-1 | Xinjiang | 277 | JHD09 | Xinjiang |
| 50 | 2000(125)/4-2-2 | Xinjiang | 164 | 2013(256)/1-2-3 | Xinjiang | 278 | 2012J176 | Xinjiang |
| 51 | 99AR142-1 | Xinjiang | 165 | 2013(256)/4-1-1 | Xinjiang | 279 | LFM1512 | Shandong |
| 52 | 99AR144-1 | Xinjiang | 166 | 2014(6)/9-1-2 | Xinjiang | 280 | LFM1430 | Shandong |
| 53 | 2009(6)/8-1-3 | Xinjiang | 167 | 2014(13)/2-2-1 | Xinjiang | 281 | 99-5091 | Xinjiang |
| 54 | 2009(67)/3-3-1 | Xinjiang | 168 | 2014(13)/5-1-2 | Xinjiang | 282 | CA12107 | Beijing |
| 55 | 2009(126)/10-2-1 | Xinjiang | 169 | 2014(13)/5-1-5 | Xinjiang | 283 | GZ1815 | Beijing |
| 56 | 2009(172)/2-2-1 | Xinjiang | 170 | 2014(14)/1-1-1 | Xinjiang | 284 | GZ1820 | Beijing |
| 57 | 2010(27)/6-5-1 | Xinjiang | 171 | 2014(14)/6-1-2 | Xinjiang | 285 | CA13012 | Beijing |
| 58 | 2010(48)/1-2-1 | Xinjiang | 172 | 2014(17)/10-1-2 | Xinjiang | 286 | CA16125 | Beijing |
| 59 | 2010(60)/4-4-3 | Xinjiang | 173 | 2014(20)/8-1-2 | Xinjiang | 287 | CA17097-1 | Beijing |
| 60 | 2010(101)/4-2-3 | Xinjiang | 174 | 2014(20)/11-2-1 | Xinjiang | 288 | 16GZ1612 | Beijing |
| 61 | 2010(112)/6-2-2 | Xinjiang | 175 | 2014(35)/6-1-2 | Xinjiang | 289 | Tangshan 6898 | Hebei |
| 62 | 2010(126)/3-1-2 | Xinjiang | 176 | 2014(37)/5-3-1 | Xinjiang | 290 | Jimai 24 | Hebei |
| 63 | 2010(162)/8-2-2 | Xinjiang | 177 | 2014(71)/3-3-1 | Xinjiang | 291 | Jimai 26 | Hebei |
| 64 | 2010(229)/1-2-2 | Xinjiang | 178 | 2014(73)/4-1-1 | Xinjiang | 292 | Jimai 31 | Hebei |
| 65 | 2011(65)/1-1-1 | Xinjiang | 179 | 2014(73)/4-1-2 | Xinjiang | 293 | Han 5316 | Hebei |
| 66 | 2011(70)/4-1-4 | Xinjiang | 180 | 2014(75)/8-2-1 | Xinjiang | 294 | Han 6172 | Hebei |
| 67 | 2011(275)/2-1-1 | Xinjiang | 181 | 2014(75)/8-2-2 | Xinjiang | 295 | Heniang 12 | Hebei |
| 68 | 2012(27)/7-2-4 | Xinjiang | 182 | 2014(75)/15-1-2 | Xinjiang | 296 | Shixin 618 | Hebei |
| 69 | 2012(27)/7-6-1 | Xinjiang | 183 | 2014(85)/2-2-1 | Xinjiang | 297 | Liangxing 66 | Hebei |
| 70 | 2012(41)/1-2-2 | Xinjiang | 184 | 2014(86)/5-2-3 | Xinjiang | 298 | Liangxing 99 | Hebei |
| 71 | 2012(80)/10-1-1 | Xinjiang | 185 | 2014(94)/2-2-2 | Xinjiang | **299** | **Jimai 17** | Shandong |
| 72 | 2012(82)/7-4-3 | Xinjiang | 186 | 2014(95)/1-2-1 | Xinjiang | **300** | **Jimai 19** | Shandong |
| 73 | 2012(84)/3-3-4 | Xinjiang | 187 | 2014(123)/1-1-1 | Xinjiang | **301** | **Jimai 20** | Shandong |
| 74 | 2012(84)/3-5-2 | Xinjiang | 188 | 2014(123)/1-2-1 | Xinjiang | **302** | **Jimai 21** | Shandong |
| 75 | 2012(103)/3-2-2 | Xinjiang | 189 | 2014(131)/1-2-1 | Xinjiang | **303** | **Jimai 22** | Shandong |
| 76 | 2012(104)/3-1-2 | Xinjiang | 190 | 2014(135)/2-1-3 | Xinjiang | 304 | Jimai 23 | Shandong |
| 77 | 2012(107)/8-1-4 | Xinjiang | 191 | 2014(151)/5-2-3 | Xinjiang | 305 | Jimai 44 | Shandong |
| 78 | 2012(111)/5-2-6 | Xinjiang | 192 | 2014(156)/2-1-3 | Xinjiang | 306 | Jimai 229 | Shandong |
| 79 | 2012(162)/4-1-3 | Xinjiang | 193 | 2014(174)/2-1-1 | Xinjiang | 307 | Yannong 19 | Shandong |
| 80 | 2012(214)/2-1-1 | Xinjiang | 194 | 2014(180)/2-2-3 | Xinjiang | 308 | Taishan 241 | Shandong |
| 81 | 2012(17)/3-1-4 | Xinjiang | 195 | 2014(184)/2-2-2 | Xinjiang | 309 | Yannong 173 | Shandong |
| 82 | 2012(41)/1-2-2 | Xinjiang | 196 | 2014(184)/2-3-2 | Xinjiang | 310 | Shannong 22 | Shandong |
| 83 | 2012(68)/8-1-1 | Xinjiang | 197 | 2014(186)/2-2-1 | Xinjiang | 311 | Yan 1212 | Shandong |
| 84 | 2012(80)/10-1-3 | Xinjiang | 198 | 2014(191)/3-2-1 | Xinjiang | 312 | Yumai 34 | Henan |
| 85 | 2012(82)/7-5-1 | Xinjiang | 199 | 2014(191)/3-2-3 | Xinjiang | 313 | Gaocheng 8901 | Henan |
| 86 | 2012(202)/4-1-2 | Xinjiang | 200 | 2014(194)/14-2-1 | Xinjiang | 314 | Zheng 366 | Henan |
| 87 | 2013(27)/8-5-1 | Xinjiang | 201 | 2014(197)/1-2-2 | Xinjiang | 315 | Zhengmai 379 | Henan |
| 88 | 2013(206)/2-1-1 | Xinjiang | 202 | 2014(199)/1-2-1 | Xinjiang | 316 | Zhengmai 7698 | Henan |
| 89 | 2011(70)/4-1-4 | Xinjiang | 203 | 2014(200)/1-1-3 | Xinjiang | 317 | Zhengmai 9023 | Henan |
| 90 | 2012(67)/9-1-2 | Xinjiang | 204 | 2014(205)/10-1-2 | Xinjiang | 318 | Zhoumai 18 | Henan |
| 91 | 2013(37)/5-4-1 | Xinjiang | 205 | 2014(205)/10-2-2 | Xinjiang | 319 | Zhoumai 19 | Henan |
| 92 | 2013(62)/2-5-1 | Xinjiang | 206 | 2014(214)/2-1-2 | Xinjiang | 320 | Zhoumai 21 | Henan |
| 93 | 2013(69)/4-1-2 | Xinjiang | 207 | 2014(229)/7-1-1 | Xinjiang | 321 | Zhoumai 22 | Henan |
| 94 | 2013(85)/2-1-1 | Xinjiang | 208 | 2014(230)/7-2-2 | Xinjiang | 322 | Zhoumai 27 | Henan |
| 95 | 2013(115)/1-5-1 | Xinjiang | 209 | 2014(233)/2-1-1 | Xinjiang | 323 | Zhoumai 28 | Henan |
| 96 | 2013(123)/1-1-1 | Xinjiang | 210 | 2014(235)/5-1-1 | Xinjiang | 324 | Zhoumai 30 | Henan |
| 97 | 2013(148)/4-1-2 | Xinjiang | 211 | 2013(28)/8-1-1 | Xinjiang | 325 | Zhoumai 32 | Henan |
| 98 | 2013(154)/2-3-1 | Xinjiang | 212 | 2013(39)/4-1-1 | Xinjiang | 326 | Fengdecunmai 5 | Henan |
| 99 | 2013(154)/4-1-3 | Xinjiang | 213 | 2013(39)/5-2-2 | Xinjiang | 327 | Guan 35 | Henan |
| 100 | 2013(178)/1-3-1 | Xinjiang | 214 | 2013(39)/5-5-3 | Xinjiang | 328 | Aikang 58 | Henan |
| 101 | 2013(223)/5-3-1 | Xinjiang | 215 | 2013(39)/11-4-1 | Xinjiang | 329 | Xiaoyan 22 | Hebei |
| 102 | 2013(227)/1-1-1 | Xinjiang | 216 | 2013(39)/15-1-1 | Xinjiang | **330** | **Xiaoyan 54** | Hebei |
| 103 | 2013(227)/1-1-2 | Xinjiang | 217 | 2013(39)/15-3-2 | Xinjiang | 331 | Kemai 1 | Beijing |
| 104 | 2013(230)/11-3-1 | Xinjiang | 218 | FZ-35/2 | Xinjiang | 332 | Xinong 979 | Shanxi |
| 105 | 2013(243)/5-2-1 | Xinjiang | 219 | FZ-46/5 | Xinjiang | 333 | Shinuan 02-1 | Hebei |
| 106 | 2013(247)/5-2-1 | Xinjiang | 220 | FZ-48/2 | Xinjiang | 334 | Zhongyou 206 | Beijing |
| 107 | 2013(248)/5-1-1 | Xinjiang | 221 | FZ-50/4 | Xinjiang | 335 | Zhongyou 9507 | Beijing |
| 108 | 2013(13)/4-1-1 | Xinjiang | 222 | FZ-59/1 | Xinjiang | 336 | Y119 | Beijing |
| 109 | 2013(23)/4-1-1 | Xinjiang | 223 | YY-2/1 | Xinjiang | 337 | Jing 411 | Beijing |
| 110 | 2013(27)/8-4-1 | Xinjiang | 224 | YY-22/4 | Xinjiang | 338 | 03-6118 | Xinjiang |
| 111 | 2013(27)/8-5-1 | Xinjiang | 225 | YY-57/3 | Xinjiang | 339 | compton/yangmai 158 | Jiangsu |
| 112 | 2013(58)/7-2-1 | Xinjiang | 226 | YY-58/1 | Xinjiang | 340 | Libellula | Italy |
| 113 | 2013(69)/4-1-1 | Xinjiang | 227 | YY-59/1 | Xinjiang | 341 | Strampellula | Italy |
| 114 | 2013(79)/3-1-1 | Xinjiang | 228 | YY-84/2 | Xinjiang |  |  |  |

Bold materials indicate those that are duplicated in Table S2. “A(B)/C” is the naming convention for advanced breeding lines. In this format, A indicates the year of hybridization, B represents the hybrid combination, and C refers to the specific breeding line.

Table S2 The names of 200 accessions in the validation panel

| Code | Name | Code | Name |
| --- | --- | --- | --- |
| DZ001 | Hongzhitou 10 | DZ312 | Heisuidongmai |
| **DZ028** | **Yinong 18** | DZ313 | Fengchan 3 |
| DZ029 | Yinong 19 | DZ314 | Shijiazhuang 54 |
| DZ030 | Yinong 20 | DZ317 | Jinmai 22 |
| DZ031 | Yinong 21 | DZ320 | Jingzuo 210 |
| DZ032 | Kuidong 4 | DZ322 | F42-7 |
| DZ034 | Kuihua 2 | DZ326 | Duli |
| DZ039 | 80-453 | DZ327 | Xiusidun |
| DZ040 | 85(1) | DZ329 | Botre |
| DZ041 | 86(46)/0-2-5-3-1 | DZ333 | Quzishi21323 |
| DZ043 | 87YF5 | DZ337 | 93-410 |
| DZ044 | 89(100) | DZ348 | 92-5 |
| DZ045 | 89-(20)-1-2 | DZ356 | Lanketa |
| DZ046 | 89(34) | DZ358 | Athas66 |
| DZ047 | 89(35) | DZ370 | Lanlixiaomai |
| DZ048 | 89-117 | DZ384 | Xiaoyan 54 |
| DZ049 | 89-20/2 | DZ386 | Zhongyin 85 |
| DZ050 | 89-44 | DZ411 | Shi 02-6207 |
| DZ051 | 89-813 | DZ419 | Bainong 68 |
| DZ052 | 91(28) | DZ444 | Lankao 906-4 |
| DZ053 | 91-8 | DZ445 | 5R624（Lr4/C9355） |
| DZ054 | 96254-1 | DZ447 | 5R623 |
| DZ055 | 99-5019 | DZ448 | 5R619（Lr38） |
| DZ056 | 0118 | DZ453 | Zhou 8425B |
| DZ058 | 05/2111 | DZ462 | Shi 02-5289 |
| DZ060 | Xindong 39 | DZ465 | 035303 |
| DZ061 | 06/6155 | DZ467 | 0138 |
| DZ062 | D04-L4 | DZ468 | Huaimai 0208 |
| DZ064 | Jintianshan 8 | DZ469 | Nongda 3291 |
| DZ068 | 98(110)/5-3-1 | DZ473 | Keyi 4058 |
| DZ069 | 98(101)/13-3 | DZ478 | Shannong 2149 |
| DZ071 | 96(60)/3-1-3 | DZ480 | CA0391-1 |
| DZ076 | 98(111)/5-2-2 | DZ487 | Shan 253 |
| DZ086 | 98(21)/2-2-3 | DZ494 | Jingdong 8 |
| DZ087 | 99(55)/5-2 | DZ500 | Shijiazhuang 8 |
| DZ089 | 98(78)/9-3-1 | DZ501 | Shimai 15 |
| DZ093 | 98(83)/2-1-1 | DZ506 | Shi 4185 |
| DZ095 | 98(107)/1-3-1 | DZ507 | Heng 5229 |
| DZ098 | 98(107)/3-1-3 | DZ515 | Zheng 9023 |
| DZ101 | 98(75)/2-1-2 | **DZ519** | **Jimai 17** |
| DZ111 | 98(76)/2-2-3 | **DZ520** | **Jimai 19** |
| DZ117 | 97(98)/1-1-3 | **DZ521** | **Jimai 20** |
| DZ118 | 97(83)/1-1-1 | **DZ522** | **Jimai 21** |
| DZ123 | 97(97)/4-2-3 | **DZ523** | **Jimai 22** |
| DZ130 | 97(24)/5-1-1 | DZ529 | Lunxuan 987 |
| DZ143 | 98(111)/5-2-1 | DZ536 | Luyuan 502 |
| DZ145 | 98(114)/1-1-1 | DZ537 | Yanda 1885 |
| DZ146 | 98(124)2-2-2 | DZ538 | Fengshou |
| DZ152 | 74-25/luofulin 10 | DZ539 | Beinong 2 |
| DZ153 | 75-149/80-25 | DZ541 | Zhongmai 9 |
| DZ154 | 75-144/Hengshui 8116 | DZ547 | Linfen 6010 |
| DZ155 | 75-144/Fengshou | DZ560 | Hengshui 8116 |
| DZ158 | Banong 1403 | DZ565 | Shan 7859 |
| DZ159 | [(170/Jingzuo210) ×80-25] F5 | DZ566 | Jinnong 207 |
| DZ168 | (80A×Gaokang1) F1/(Dao×Jiu)/75-149/Jinan 13 | DZ568 | You8134 |
| DZ169 | (Dao×Jiu)//75-149×9-142/506/80A-2/Jingai 1 | DZ570 | Henong 326 |
| DZ172 | (Xindong 14×Lov10/somon/75-144×Hengshui 8116)F2 | DZ573 | Jinan13 |
| DZ175 | 75-149/(Y83-E4/Honggou×2407) | DZ575 | Jinanhe124 |
| DZ182 | 80-25 | DZ592 | Rusattea |
| DZ185 | 2001(38)/1-2-6 | DZ593 | Asosan |
| DZ186 | 2002(85)/4-1-2 | DZ597 | Atom MY4310 |
| DZ190 | 2001(43)/2-1-4 | DZ598 | Arha MY4270 |
| DZ193 | 2003(28)/4-3-1 | DZ599 | Atgton MY4168 |
| DZ195 | 2003(29)/3-2-4 | DZ600 | Knteh MY6280 |
| DZ196 | 2003(36)/6-3-2 | DZ601 | F181-3 MY5585 |
| DZ199 | 2003(76)/3-11-2 | DZ602 | MY4348 Attnla |
| DZ203 | 2002(41)/4-4-3 | DZ649 | Nongda 212 |
| DZ210 | 2000(115)/2-1-2 | DZ659 | Zhongmai 175 |
| DZ211 | 2000(139)/1-1-1 | DZ680 | Nongda 3488 |
| DZ214 | 2000(8)/1-3-2 | DZ681 | Yumai 34 |
| DZ215 | 2000(16)/2-2-2 | DZ682 | Wanmai 52 |
| DZ217 | 2001(43)/9-2-3 | DZ683 | Xinmai 18 |
| DZ218 | Kendong 03(10) | DZ684 | Yanzhan 4110 |
| DZ221 | Kendong 01(38) | DZ704 | Xiaobaidongmai |
| DZ224 | 2005(1)/7-4-6 | DZ708 | Kuchebaidongmai |
| DZ232 | 2005(62)/15-4-3 | DZ717 | Kayimuhong |
| DZ234 | 2005(65)/7-2-1 | DZ726 | Kadong 4 |
| DZ242 | 2012Hanqu3 | DZ728 | Youpilaina 1 |
| DZ244 | 2012Hanqu5 | DZ729 | Xiaoe 186 |
| DZ247 | 2012Hanqu8 | DZ731 | Aodesa6 |
| DZ250 | 2012Hanqu11 | DZ732 | Xinwukelan 83 |
| DZ255 | 2012Wanqu4 | DZ733 | Xinwukelan 84 |
| DZ256 | 2012Wanqu5 | DZ734 | Aodesa3 |
| DZ257 | 2012Wanqu6 | DZ735 | Wumang 1 |
| DZ259 | 2012Wanqu8 | DZ737 | Afuleer |
| DZ264 | 2012Zaosheng 2(95022-8-1-7) | DZ738 | Zaoyangmai |
| DZ265 | 2006(114)/5-10-2 | DZ740 | Aoweisite |
| DZ266 | 2007Lun2/6-3-1 | DZ742 | Shanqian 2 |
| DZ269 | 2007(6)/13-5-2 | DZ743 | Gaojiasuo |
| DZ270 | 2007(8)/26-2-2 | DZ745 | Huabei 187 |
| DZ271 | 2007(16)/25-5-2 | DZ746 | Huabei 497 |
| DZ272 | 2007(26)/6-1-3 | DZ747 | Beijing 6 |
| DZ274 | 2007(32)/7-4-3 | DZ748 | Beijing 7 |
| DZ275 | 2007(37)/20-1-3 | DZ752 | Jinan 4 |
| DZ277 | 2007(44)/13-2 | DZ754 | Baiyoubao |
| DZ290 | Jihe 124 | DZ758 | Gongnong19 |
| DZ291 | Rusaika | DZ759 | Luofulin 10 |
| DZ292 | Aigton MY4168 | DZ760 | Luofulin 18 |
| DZ299 | Mianyang 31 | DZ761 | Luofulin 13 |
| DZ306 | Kuiyinnan 4 | DZ762 | Cangdong 5 |

Bold materials indicate those that are duplicated in Table S1

Table S3 The primer sequences for the KASP assay

| Marker | Probe | Sequence (5'-3') |
| --- | --- | --- |
| *1D_415401424* | FAM | GAAGGTGACCAAGTTCATGCTGGCGTCAGATCATGCCACTT |
| *1D_415401424* | HEX | GAAGGTCGGAGTCAACGGATTGGCGTCAGATCATGCCACTC |
| *1D_415401424* | Common | CGCAGCTTCCATACTGGCTA |
| *1D_415704212* | FAM | GAAGGTGACCAAGTTCATGCTTGATAGTGAGGGATAGTAGGCAA |
| *1D_415704212* | HEX | GAAGGTCGGAGTCAACGGATTTGATAGTGAGGGATAGTAGGCAG |
| *1D_415704212* | Common | GCCTACAACCATTGTGCCCT |
| *1D_412160361* | FAM | GAAGGTGACCAAGTTCATGCTCGGTAAGAAGGAGCGGCC |
| *1D_412160361* | HEX | GAAGGTCGGAGTCAACGGATTCGGTAAGAAGGAGCGGCA |
| *1D_412160361* | Common | CACATTCTTCGGGTGCAGAT |
| *1D_413224542* | FAM | GAAGGTGACCAAGTTCATGCTGCAAATGCAGCAACCGCA |
| *1D_413224542* | HEX | GAAGGTCGGAGTCAACGGATTGCAAATGCAGCAACCGCG |
| *1D_413224542* | Common | TGGATTCTCGCCCTGTGAAG |
| *1D_413729079* | FAM | GAAGGTGACCAAGTTCATGCTGTGCAGTTGGGTCGAGATTTA |
| *1D_413729079* | HEX | GAAGGTCGGAGTCAACGGATTGTGCAGTTGGGTCGAGATTTT |
| *1D_413729079* | Common | TGCGGATTGCTCGGTAAGTG |
| *1D_414451522* | FAM | GAAGGTGACCAAGTTCATGCTCCGTCCTGATACTAGTATTATGGAT |
| *1D_414451522* | HEX | GAAGGTCGGAGTCAACGGATTCCGTCCTGATACTAGTATTATGGAG |
| *1D_414451522* | Common | GCCAACGTCCAATCACCAGA |
| *1A_236720351* | FAM | GAAGGTGACCAAGTTCATGCTTCCTCATCCATTCCTAATGCAA |
| *1A_236720351* | HEX | GAAGGTCGGAGTCAACGGATTTCCTCATCCATTCCTAATGCAG |
| *1A_236720351* | Common | TTGTGGAGGATGTTCGGCAA |
| *1B_9992236* | FAM | GAAGGTGACCAAGTTCATGCTTCCAGAGCTTCTTGAGGGTTC |
| *1B_9992236* | HEX | GAAGGTCGGAGTCAACGGATTTCCAGAGCTTCTTGAGGGTTT |
| *1B_9992236* | Common | GAACATGGTCCCTCATGGCA |
| *1D_19807137* | FAM | GAAGGTGACCAAGTTCATGCTGGCAAGCATCTCCGGCTG |
| *1D_19807137* | HEX | GAAGGTCGGAGTCAACGGATTGGCAAGCATCTCCGGCTA |
| *1D_19807137* | Common | CCGTTCACACGCCATTCTTC |
| *1B_53066005* | FAM | GAAGGTGACCAAGTTCATGCTGCATTAGCCTACGATGAAACTGC |
| *1B_53066005* | HEX | GAAGGTCGGAGTCAACGGATTGCATTAGCCTACGATGAAACTGA |
| *1B_53066005* | Common | AATCGGCATCACAACATGGC |
| *1A_12815998* | FAM | GAAGGTGACCAAGTTCATGCTTGAGCAAATCTCAGGGACAT |
| *1A_12815998* | HEX | GAAGGTCGGAGTCAACGGATTTGAGCAAATCTCAGGGACAC |
| *1A_12815998* | Common | CAACCTCTCGACGTGGGTAG |

Table S4 Protein quality traits of two extreme accessions

| Cultivar | Grain protein | Flour protein | Wet gluten content | Dry gluten content | Gluten index | Zeleny sedimentation value |
| --- | --- | --- | --- | --- | --- | --- |
| Hongzhitou | 17.10 | 14.66 | 42.41 | 13.78 | 43.78 | 32.00 |
| Huaimai0208 | 13.80 | 10.20 | 29.82 | 10.20 | 91.94 | 18.00 |

Table S5 The gene IDs and primer sequences used in real-time quantitative polymerase chain reaction (RT-qPCR)

| Gene ID | Primer F (5'-3') | Primer R (5'-3') |
| --- | --- | --- |
| *TraesCS6B02G230300.2* | GATCAATGGCGTGCCAATCC | AACAAGACGGGTAAAGGGCA |
| *TraesCS1A02G137700.2* | CATCCGTCAGCCTCTCATCC | TTACAGTGCAACGGCTCCTT |
| *TraesCS1B02G068200.1* | TGCTCAAGAAGGAAGGCAGG | AGTCACCAAATCCAGCGTCT |
| *TraesCS1B02G068500.2* | TACCCTGGCTCATCTGGTCA | CCGTGAGAAAGCTTGTGTGC |
| *TraesCS1D02G000700.1* | CGTCGTGCACGCCATTATTT | CACCCATGTCCCTATCACCG |
| TraesCS1D02G000800.2 | TTGACAAGGGCAACGGTACT | CCAGCCACGGTGTCTTCAT |
| *TraesCS1D02G001200.1* | TCGACCCTAGCGGCCAAGTA | TGGGATAATGGCTGTTGGAGCTG |
| *TraesCS1D02G001300.1* | ATTGGGCCAGCACTCTATCC | CATGCTACCAAATGGTGCAGTAAT |
| *TraesCS1D02G009400.1* | CAGCAGCTAAACCCATGCAA | CCTAGCAAGACGTTGCGACA |
| *TraesCS1D02G018300.1* | CATCGCCGTCATCATCTCCA | GGATCATTCCGACGAACAGC |
| *TraesCS1B02G286800.1* | ATCACCGAACGAGACACGAAG | AATCTTCCTCAGCCTCCCGT |
| *TRAESCS1D02G323100* | ATGGCGCCGCCTCTC | TGGCCTGGTCGAAATCGAAG |
| *TraesCS1A02G100200* | CATGGGCTGGTTGTCATTGC | TCCTCTTCGTTACCTCTTGTTCA |
| *Actin* | GGAAAAGTGCAGAGAGACACG | TACAGTGTCTGGATCGGTGGT |

Table S6 Analysis of variance for protein quality traits in 341 winter wheats

| Source of variance | *df* | Sum of squares | | | | | |
| --- | --- | --- | --- | --- | --- | --- | --- |
|  |  | Grain protein content | Flour protein content | Wet gluten content | Dry gluten content | Gluten index | Zeleny sedimentation value |
| genotypes | 340 | 2.5*** | 2*** | 64*** | 10*** | 469*** | 58*** |
| Environments | 1 | 1841.3*** | 1491.8*** | 14430*** | 2802*** | 2582*** | 7488*** |
| years | 1 | 1475.5*** | 1155.9*** | 24262*** | 4542*** | 3259*** | 196*** |
| genotypes х environments | 339 | 0.4 | 0.4 | 15 | 3 | 133*** | 8 |
| genotypes х years | 339 | 0.4 | 0.3 | 11 | 2 | 120** | 5 |
| Residual | 332 | 0.4 | 0.3 | 18 | 3 | 93 | 10 |

Table S7 Significant SNPs associated with protein quality traits by genome-wide association study using BLUP values

| Trait | Marker | Chromosome | Position(bp) | *P*-value | *R^2^*(%) |
| --- | --- | --- | --- | --- | --- |
| Grain protein content | *1A_57737689* | 1A | 57737689 | 6.41E-05 | 5.86 |
|  | *2A_517926919* | 2A | 517926919 | 3.94E-05 | 6.28 |
|  | *5A_455140284* | 5A | 455140284 | 8.93E-05 | 5.65 |
|  | *5A_455461352* | 5A | 455461352 | 9.12E-05 | 5.64 |
|  | *7A_647143478* | 7A | 647143478 | 5.13E-05 | 6.00 |
|  | *2B_110138413* | 2B | 110138413 | 5.62E-05 | 5.94 |
|  | *2B_749404797* | 2B | 749404797 | 9.97E-05 | 5.58 |
|  | *3B_27229830* | 3B | 27229830 | 7.25E-05 | 5.78 |
|  | *5B_631618808* | 5B | 631618808 | 4.74E-05 | 6.22 |
|  | *5D_67352562* | 5D | 67352562 | 5.49E-05 | 5.96 |
|  | *5D_73538903* | 5D | 73538903 | 5.40E-05 | 5.97 |
|  | *5D_129671910* | 5D | 129671910 | 5.43E-05 | 5.96 |
|  | *5D_147564473* | 5D | 147564473 | 5.43E-05 | 5.96 |
|  | *5D_230426321* | 5D | 230426321 | 5.44E-05 | 5.96 |
|  | *5D_255119942* | 5D | 255119942 | 4.80E-05 | 6.04 |
|  | *5D_273085178* | 5D | 273085178 | 4.86E-05 | 6.03 |
|  | *5D_275210924* | 5D | 275210924 | 4.86E-05 | 6.03 |
|  | *5D_290788735* | 5D | 290788735 | 5.99E-05 | 5.93 |
|  | *5D_291338264* | 5D | 291338264 | 3.45E-05 | 6.25 |
|  | *6D_410156938* | 6D | 410156938 | 9.05E-05 | 5.64 |
|  | *7D_102587780* | 7D | 102587780 | 1.94E-05 | 6.61 |
| Flour protein content | *4A_626115009* | 4A | 626115009 | 8.23E-05 | 5.70 |
|  | *7A_647143478* | 7A | 647143478 | 4.34E-05 | 6.10 |
|  | *6D_410156938* | 6D | 410156938 | 5.15E-05 | 6.00 |
| Wet gluten content | *3A_11830020* | 3A | 11830020 | 4.16E-05 | 6.13 |
|  | *4B_326456149* | 4B | 326456149 | 1.46E-05 | 6.90 |
|  | *4B_559645261* | 4B | 559645261 | 6.14E-05 | 5.89 |
|  | *5B_134937177* | 5B | 134937177 | 6.63E-05 | 5.84 |
|  | *5B_221713044* | 5B | 221713044 | 8.33E-05 | 5.70 |
|  | *1D_412136490* | 1D | 412136490 | 8.71E-05 | 5.68 |
|  | *1D_412160361* | 1D | 412160361 | 7.07E-05 | 5.80 |
|  | *1D_412196822* | 1D | 412196822 | 7.87E-05 | 5.74 |
|  | *1D_412227592* | 1D | 412227592 | 2.78E-05 | 6.39 |
|  | *1D_413292355* | 1D | 413292355 | 5.97E-05 | 5.91 |
|  | *1D_413729079* | 1D | 413729079 | 6.52E-05 | 5.85 |
|  | *1D_414286643* | 1D | 414286643 | 2.97E-05 | 6.37 |
|  | *1D_414493743* | 1D | 414493743 | 5.74E-05 | 5.93 |
|  | *1D_414500837* | 1D | 414500837 | 5.98E-05 | 5.91 |
|  | *1D_414573947* | 1D | 414573947 | 7.51E-05 | 5.76 |
|  | *1D_414708956* | 1D | 414708956 | 7.41E-05 | 5.77 |
|  | *1D_415213648* | 1D | 415213648 | 6.82E-05 | 5.92 |
|  | *1D_415401424* | 1D | 415401424 | 3.11E-05 | 6.32 |
|  | *1D_415466562* | 1D | 415466562 | 6.18E-05 | 5.89 |
|  | *1D_416033546* | 1D | 416033546 | 7.93E-05 | 5.73 |
| Gluten index | *1A_12815998* | 1A | 12815998 | 5.70E-05 | 6.04 |
|  | *1A_20940731* | 1A | 20940731 | 7.00E-05 | 5.81 |
|  | *3A_166183080* | 3A | 166183080 | 7.13E-05 | 5.80 |
|  | *3A_201395455* | 3A | 201395455 | 9.72E-05 | 5.60 |
|  | *1B_558555488* | 1B | 558555488 | 4.37E-05 | 6.10 |
|  | *1B_558573212* | 1B | 558573212 | 5.02E-05 | 6.02 |
|  | *1B_558931435* | 1B | 558931435 | 5.45E-05 | 5.98 |
|  | *1B_558975526* | 1B | 558975526 | 5.02E-05 | 6.02 |
|  | *1B_559539573* | 1B | 559539573 | 2.06E-05 | 6.79 |
|  | *1B_561268092* | 1B | 561268092 | 4.41E-05 | 5.05 |
|  | *2B_5043248* | 2B | 5043248 | 3.22E-05 | 6.39 |
|  | *5B_239318081* | 5B | 239318081 | 3.34E-05 | 6.27 |
|  | *5B_548363217* | 5B | 548363217 | 3.98E-06 | 7.61 |
|  | *1D_408533013* | 1D | 408533013 | 1.55E-05 | 6.75 |
|  | *1D_411177352* | 1D | 411177352 | 6.53E-05 | 5.85 |
|  | *1D_411192168* | 1D | 411192168 | 5.80E-06 | 7.38 |
|  | *1D_411237031* | 1D | 411237031 | 6.51E-06 | 7.30 |
|  | *1D_411295558* | 1D | 411295558 | 3.19E-06 | 7.75 |
|  | *1D_411320325* | 1D | 411320325 | 9.62E-07 | 8.56 |
|  | *1D_411366902* | 1D | 411366902 | 3.27E-05 | 6.29 |
|  | *1D_411374953* | 1D | 411374953 | 1.67E-05 | 6.71 |
|  | *1D_411404496* | 1D | 411404496 | 2.19E-05 | 6.66 |
|  | *1D_411727551* | 1D | 411727551 | 2.87E-06 | 7.82 |
|  | *1D_412023762* | 1D | 412023762 | 2.30E-06 | 7.96 |
|  | *1D_412031073* | 1D | 412031073 | 1.65E-06 | 8.17 |
|  | *1D_412072331* | 1D | 412072331 | 4.49E-06 | 7.54 |
|  | *1D_412092560* | 1D | 412092560 | 4.53E-06 | 7.53 |
|  | *1D_412136490* | 1D | 412136490 | 2.58E-06 | 7.95 |
|  | *1D_412160361* | 1D | 412160361 | 5.10E-06 | 7.46 |
|  | *1D_412178540* | 1D | 412178540 | 2.28E-05 | 6.51 |
|  | *1D_412181832* | 1D | 412181832 | 1.51E-05 | 6.78 |
|  | *1D_412196822* | 1D | 412196822 | 2.67E-05 | 6.43 |
|  | *1D_412227592* | 1D | 412227592 | 5.44E-07 | 8.89 |
|  | *1D_412288344* | 1D | 412288344 | 1.83E-05 | 6.65 |
|  | *1D_412495297* | 1D | 412495297 | 1.00E-05 | 7.18 |
|  | *1D_413224542* | 1D | 413224542 | 4.69E-07 | 8.98 |
|  | *1D_413292355* | 1D | 413292355 | 3.79E-06 | 7.64 |
|  | *1D_413311188* | 1D | 413311188 | 4.05E-06 | 7.60 |
|  | *1D_413729079* | 1D | 413729079 | 2.62E-06 | 7.88 |
|  | *1D_413934525* | 1D | 413934525 | 9.34E-06 | 7.07 |
|  | *1D_414144652* | 1D | 414144652 | 2.43E-06 | 7.93 |
|  | *1D_414286643* | 1D | 414286643 | 4.15E-06 | 7.59 |
|  | *1D_414451522* | 1D | 414451522 | 9.46E-06 | 7.07 |
|  | *1D_414493743* | 1D | 414493743 | 1.93E-06 | 8.08 |
|  | *1D_414500837* | 1D | 414500837 | 1.42E-06 | 8.27 |
|  | *1D_414573947* | 1D | 414573947 | 4.87E-06 | 7.49 |
|  | *1D_414708956* | 1D | 414708956 | 3.51E-06 | 7.69 |
|  | *1D_415059649* | 1D | 415059649 | 1.82E-05 | 6.65 |
|  | *1D_415126157* | 1D | 415126157 | 4.06E-06 | 7.60 |
|  | *1D_415158238* | 1D | 415158238 | 6.00E-05 | 5.90 |
|  | *1D_415213648* | 1D | 415213648 | 3.24E-06 | 7.90 |
|  | *1D_415290059* | 1D | 415290059 | 7.47E-06 | 7.21 |
|  | *1D_415298532* | 1D | 415298532 | 2.90E-06 | 7.82 |
|  | *1D_415401424* | 1D | 415401424 | 2.66E-06 | 7.87 |
|  | *1D_415459431* | 1D | 415459431 | 7.36E-06 | 7.22 |
|  | *1D_415466562* | 1D | 415466562 | 9.83E-06 | 7.04 |
|  | *1D_415586977* | 1D | 415586977 | 4.19E-06 | 7.58 |
|  | *1D_415645580* | 1D | 415645580 | 3.86E-06 | 7.63 |
|  | *1D_415704212* | 1D | 415704212 | 3.54E-06 | 7.69 |
|  | *1D_415749624* | 1D | 415749624 | 4.66E-06 | 7.51 |
|  | *1D_415766966* | 1D | 415766966 | 8.00E-08 | 10.73 |
|  | *1D_416033546* | 1D | 416033546 | 5.19E-06 | 7.44 |
|  | *1D_416081310* | 1D | 416081310 | 6.16E-06 | 7.34 |
|  | *1D_416093683* | 1D | 416093683 | 3.21E-06 | 7.75 |
|  | *1D_416101167* | 1D | 416101167 | 1.04E-05 | 7.00 |
|  | *1D_416212526* | 1D | 416212526 | 1.22E-05 | 6.90 |
| Zeleny sedimentation value | *1A_9574254* | 1A | 9574254 | 1.73E-05 | 5.61 |
|  | *1A_12815998* | 1A | 12815998 | 6.74E-05 | 5.92 |
|  | *1A_21873794* | 1A | 21873794 | 8.45E-06 | 6.03 |
|  | *1A_31178918* | 1A | 31178918 | 5.30E-05 | 4.94 |
|  | *1A_33855698* | 1A | 33855698 | 7.21E-05 | 4.76 |
|  | *1A_96454293* | 1A | 96454293 | 5.86E-05 | 4.89 |
|  | *1A_225520951* | 1A | 225520951 | 1.78E-05 | 6.66 |
|  | *1A_236720351* | 1A | 236720351 | 1.13E-07 | 8.67 |
|  | *1A_508723612* | 1A | 508723612 | 6.86E-05 | 5.82 |
|  | *2A_192541883* | 2A | 192541883 | 1.66E-05 | 6.71 |
|  | *2A_425759128* | 2A | 425759128 | 3.25E-05 | 5.23 |
|  | *3A_686822185* | 3A | 686822185 | 7.93E-05 | 4.73 |
|  | *4A_177632748* | 4A | 177632748 | 3.34E-05 | 5.22 |
|  | *1B_9992236* | 1B | 9992236 | 2.71E-06 | 7.86 |
|  | *1B_53066005* | 1B | 53066005 | 2.71E-06 | 7.86 |
|  | *1B_639921450* | 1B | 639921450 | 5.84E-05 | 5.92 |
|  | *4B_4564310* | 4B | 4564310 | 5.21E-05 | 6.08 |
|  | *4B_500743139* | 4B | 500743139 | 7.43E-06 | 6.11 |
|  | *4B_550381492* | 4B | 550381492 | 7.44E-05 | 4.75 |
|  | *5B_400532969* | 5B | 400532969 | 7.16E-05 | 5.80 |
|  | *5B_548363217* | 5B | 548363217 | 8.02E-05 | 5.72 |
|  | *6B_42675975* | 6B | 42675975 | 1.25E-05 | 6.92 |
|  | *6B_43515830* | 6B | 43515830 | 7.36E-06 | 7.22 |
|  | *6B_370150478* | 6B | 370150478 | 1.77E-06 | 6.98 |
|  | *1D_232760* | 1D | 232760 | 3.48E-05 | 5.20 |
|  | *1D_6299684* | 1D | 6299684 | 3.78E-05 | 5.14 |
|  | *1D_19807137* | 1D | 19807137 | 7.45E-05 | 5.77 |
|  | *1D_49579197* | 1D | 49579197 | 3.06E-05 | 6.32 |
|  | *1D_56039301* | 1D | 56039301 | 5.62E-05 | 5.94 |
|  | *4D_238181951* | 4D | 238181951 | 1.91E-07 | 8.34 |
|  | *5D_408811401* | 5D | 408811401 | 8.82E-05 | 5.66 |
|  | *7D_37949527* | 7D | 37949527 | 1.37E-05 | 5.74 |
|  | *7D_96433791* | 7D | 96433791 | 3.75E-05 | 6.87 |

Table S8 *P*-values of *t*-tests for efficacy of different alleles on protein quality traits

| Trait | Marker | Reference | Allele | | | Number of cultivars | | | *P*-value |  |  |  |
| --- | --- | --- | --- | --- | --- | --- | --- | --- | --- | --- | --- | --- |
|  |  |  |  |  |  |  |  |  | 2020EM | 2020QT | 2021EM | 2021QT |
| Grain protein content | *6D_410156938* | T | CC | TC | TT | 89 | 8 | 242 | 0.251 | 0.000 | 0.007 | 0.043 |
|  | *2A_517926919* | G | AA | GA | GG | 185 | 18 | 129 | 0.000 | 0.003 | 0.000 | 0.006 |
|  | *3B_27229830* | G | AA | GA | GG | 273 | 8 | 58 | 0.000 | 0.008 | 0.000 | 0.001 |
|  | *5A_455461352* | A | AA | AG | GG | 268 | 14 | 57 | 0.382 | 0.104 | 0.007 | 0.006 |
| Flour protein content | *7A_647143478* | T | CC | TC | TT | 225 | 9 | 105 | 0.000 | 0.001 | 0.162 | 0.275 |
|  | *6D_410156938* | T | CC | TC | TT | 89 | 8 | 242 | 0.041 | 0.000 | 0.109 | 0.003 |
| Wet gluten content | *1D_412227592* | T | AA | TA | TT | 88 | 12 | 239 | 0.000 | 0.000 | 0.000 | 0.000 |
|  | *1D_415401424* | T | CC | TC | TT | 91 | 23 | 238 | 0.000 | 0.000 | 0.000 | 0.000 |
| Gluten index | *1D_411727551* | A | AA | AG | GG | 239 | 11 | 91 | 0.000 | 0.000 | 0.000 | 0.000 |
|  | *1D_412023762* | T | CC | TC | TT | 90 | 12 | 239 | 0.000 | 0.000 | 0.000 | 0.000 |
|  | *1D_415704212* | T | CC | TC | TT | 93 | 33 | 215 | 0.000 | 0.000 | 0.000 | 0.000 |
|  | *1D_411404496* | T | CC | TC | TT | 93 | 33 | 215 | 0.000 | 0.000 | 0.000 | 0.000 |
|  | *1D_412031073* | G | AA | GA | GG | 92 | 8 | 241 | 0.000 | 0.000 | 0.000 | 0.000 |
|  | *1D_412092560* | C | CC | CT | TT | 241 | 11 | 89 | 0.000 | 0.000 | 0.000 | 0.000 |
|  | *1D_412136490* | C | CC | CT | TT | 245 | 7 | 88 | 0.000 | 0.000 | 0.000 | 0.000 |
|  | *1D_412160361* | C | AA | CA | CC | 92 | 22 | 227 | 0.000 | 0.000 | 0.000 | 0.000 |
|  | *1D_412178540* | G | GG | GT | TT | 244 | 10 | 87 | 0.000 | 0.000 | 0.000 | 0.000 |
|  | *1D_412181832* | A | AA | AC | CC | 243 | 8 | 90 | 0.000 | 0.000 | 0.000 | 0.000 |
|  | *1D_412227592* | T | AA | TA | TT | 89 | 12 | 240 | 0.000 | 0.000 | 0.000 | 0.000 |
|  | *1D_413224542* | T | CC | TC | TT | 92 | 9 | 240 | 0.000 | 0.000 | 0.000 | 0.000 |
|  | *1D_413292355* | C | CC | CG | GG | 241 | 8 | 92 | 0.000 | 0.000 | 0.000 | 0.000 |
|  | *1D_413311188* | T | GG | TG | TT | 89 | 12 | 240 | 0.000 | 0.000 | 0.000 | 0.000 |
|  | *1D_413729079* | A | AA | AT | TT | 240 | 9 | 92 | 0.000 | 0.000 | 0.000 | 0.000 |
|  | *1D_414286643* | T | CC | TC | TT | 90 | 11 | 240 | 0.000 | 0.000 | 0.000 | 0.000 |
|  | *1D_414451522* | T | GG | TG | TT | 90 | 9 | 242 | 0.000 | 0.000 | 0.000 | 0.000 |
|  | *1D_414500837* | T | CC | TC | TT | 92 | 9 | 240 | 0.000 | 0.000 | 0.000 | 0.000 |
|  | *1D_414573947* | C | CC | CT | TT | 122 | 127 | 92 | 0.000 | 0.000 | 0.000 | 0.000 |
|  | *1D_414708956* | T | CC | TC | TT | 92 | 10 | 239 | 0.000 | 0.000 | 0.000 | 0.000 |
|  | *1D_415126157* | G | AA | GA | GG | 91 | 12 | 238 | 0.000 | 0.000 | 0.000 | 0.000 |
|  | *1D_415401424* | T | CC | TC | TT | 91 | 12 | 238 | 0.000 | 0.000 | 0.000 | 0.000 |
|  | *1D_415459431* | T | AA | TA | TT | 91 | 9 | 241 | 0.000 | 0.000 | 0.000 | 0.000 |
|  | *1D_415749624* | G | AA | GA | GG | 86 | 11 | 244 | 0.000 | 0.000 | 0.000 | 0.000 |
|  | *1D_416093683* | T | CC | TC | TT | 91 | 8 | 242 | 0.000 | 0.000 | 0.000 | 0.000 |
|  | *1D_416101167* | T | CC | TC | TT | 88 | 13 | 240 | 0.000 | 0.000 | 0.000 | 0.000 |
|  | *1D_416212526* | G | AA | GA | GG | 90 | 9 | 242 | 0.000 | 0.000 | 0.000 | 0.000 |
|  | *1D_411320325* | G | AA | GA | GG | 86 | 15 | 239 | 0.000 | 0.000 | 0.000 | 0.000 |
|  | *1D_411192168* | C | AA | CA | CC | 91 | 12 | 237 | 0.000 | 0.000 | 0.000 | 0.000 |
|  | *1D_415213648* | C | CC | CT | TT | 240 | 11 | 87 | 0.000 | 0.000 | 0.000 | 0.000 |
|  | *1B_559539573* | C | CC | CG | GG | 313 | 4 | 18 | 0.032 | 0.002 | 0.001 | 0.016 |
|  | *1A_20940731* | C | CC | CT | TT | 222 | 16 | 101 | 0.192 | 0.007 | 0.285 | 0.382 |
|  | *1B_561268092* | T | TT | TC |  | 82 | 257 |  | 0.000 | 0.000 | 0.000 | 0.000 |
|  | *1D_411295558* | G | GG | GT | TT | 237 | 12 | 90 | 0.000 | 0.000 | 0.000 | 0.000 |
|  | *1D_411366902* | T | AA | TA | TT | 91 | 12 | 236 | 0.000 | 0.000 | 0.000 | 0.000 |
|  | *1D_411374953* | G | AA | GA | GG | 92 | 12 | 235 | 0.000 | 0.000 | 0.000 | 0.000 |
|  | *1D_412072331* | G | CC | GC | GG | 90 | 10 | 239 | 0.000 | 0.000 | 0.000 | 0.000 |
|  | *1D_412196822* | G | AA | GA | GG | 88 | 11 | 240 | 0.000 | 0.000 | 0.000 | 0.000 |
|  | *1D_412288344* | C | CC | CT | TT | 241 | 10 | 88 | 0.000 | 0.000 | 0.000 | 0.000 |
|  | *1D_414493743* | C | CC | CT | TT | 239 | 13 | 87 | 0.000 | 0.000 | 0.000 | 0.000 |
|  | *1D_415059649* | G | AA | GA | GG | 86 | 14 | 239 | 0.000 | 0.000 | 0.000 | 0.000 |
|  | *1D_415290059* | C | CC | CG | GG | 241 | 8 | 90 | 0.000 | 0.000 | 0.000 | 0.000 |
|  | *1D_415298532* | A | AA | AG | GG | 240 | 10 | 89 | 0.000 | 0.000 | 0.000 | 0.000 |
|  | *1D_415466562* | G | AA | GA | GG | 90 | 11 | 238 | 0.000 | 0.000 | 0.000 | 0.000 |
|  | *1D_415586977* | T | CC | TC | TT | 90 | 8 | 241 | 0.000 | 0.000 | 0.000 | 0.000 |
|  | *1D_415645580* | T | CC | TC | TT | 89 | 10 | 240 | 0.000 | 0.000 | 0.000 | 0.000 |
|  | *1D_416081310* | C | CC | CT | TT | 242 | 7 | 90 | 0.000 | 0.000 | 0.000 | 0.000 |
| Zeleny sedimentation value | *4D_238181951* | A | AA | AT |  | 285 | 54 |  | 0.000 | 0.000 | 0.000 | 0.000 |
|  | *6B_370150478* | A | AA | AC |  | 290 | 49 |  | 0.000 | 0.000 | 0.000 | 0.000 |
|  | *1A_225520951* | G | AA | GA | GG | 19 | 54 | 266 | 0.000 | 0.000 | 0.000 | 0.000 |
|  | *1A_21873794* | C | CC | CT |  | 286 | 53 |  | 0.000 | 0.000 | 0.000 | 0.000 |
|  | *1A_236720351* | T | TC | TT |  | 58 | 281 |  | 0.000 | 0.000 | 0.000 | 0.000 |
|  | *1B_53066005* | C | AA | CA | CC | 41 | 13 | 285 | 0.000 | 0.000 | 0.000 | 0.000 |
|  | *1D_232760* | A | AA | AT |  | 284 | 54 |  | 0.000 | 0.000 | 0.000 | 0.000 |
|  | *1D_6299684* | T | TG | TT |  | 53 | 286 |  | 0.000 | 0.000 | 0.000 | 0.000 |
|  | *1B_9992236* | G | AA | GA | GG | 48 | 10 | 281 | 0.000 | 0.000 | 0.000 | 0.000 |
|  | *1D_49579197* | A | AA | AG | GG | 87 | 15 | 237 | 0.000 | 0.044 | 0.023 | 0.003 |
|  | *4B_550381492* | T | TC | TT |  | 54 | 285 |  | 0.000 | 0.000 | 0.000 | 0.000 |
|  | *6B_43515830* | A | AA | AG | GG | 288 | 9 | 42 | 0.001 | 0.011 | 0.001 | 0.001 |
|  | *7D_37949527* | G | AA | AG | GG | 288 | 9 | 42 | 0.001 | 0.011 | 0.001 | 0.001 |
|  | *1A_41516924* | T | TA | TT |  | 46 | 293 |  | 0.000 | 0.001 | 0.000 | 0.000 |
|  | *1A_96454293* | C | CC | CG |  | 285 | 54 |  | 0.000 | 0.000 | 0.000 | 0.000 |
|  | *2A_192541883* | G | AA | GA | GG | 64 | 7 | 268 | 0.005 | 0.044 | 0.038 | 0.015 |
|  | *3A_686822185* | T | TA | TT |  | 36 | 300 |  | 0.000 | 0.000 | 0.000 | 0.000 |
|  | *4A_177632748* | T | TG | TT |  | 47 | 292 |  | 0.000 | 0.000 | 0.000 | 0.000 |
|  | *5D_408811401* | T | AA | TA | TT | 47 | 31 | 261 | 0.000 | 0.000 | 0.000 | 0.000 |
|  | *1A_12815998* | T | CC | TC | TT | 75 | 13 | 250 | 0.024 | 0.006 | 0.205 | 0.000 |
|  | *1A_31178918* | G | GA | GG |  | 59 | 280 |  | 0.000 | 0.000 | 0.000 | 0.000 |
|  | *1D_19807137* | C | CC | CT | TT | 58 | 12 | 269 | 0.017 | 0.318 | 0.175 | 0.007 |
|  | *7D_96433791* | C | CC | CT | TT | 252 | 49 | 7 | 0.000 | 0.000 | 0.000 | 0.000 |
|  | *3A_65406649* | T | TC | TT |  | 47 | 292 |  | 0.000 | 0.000 | 0.000 | 0.000 |

Table S9 Haplotypes with different alleles in the blocks

| Trait | Block | SNP | Haplotype | | Number of cultivars | |
| --- | --- | --- | --- | --- | --- | --- |
|  |  |  | Superior | Inferior | Superior | Inferior |
| Gluten index | Block 1 | *1D_415059649/1D_415126157/1D_415158238/1D_415171812/1D_415213648/1D_415267964/1D_415290059/1D_415298532/1D_415401424/1D_415459431/1D_415466562* | AA/AA/TT/TT/TT/GG/GG/GG/CC/AA/AA | GG/GG/AA/CC/CC/AA/CC/AA/TT/TT/GG | 84 | 83 |
|  | Block 2 | *1D_415645580/1D_415704212/1D_415749624/1D_415766966/1D_416033546/1D_416081310/1D_416093683/1D_416101167* | CC/CC/AA/GG/GG/TT/CC/CC | TT/TT/GG/AA/AA/CC/TT/TT | 205 | 78 |
|  | Block 3 | *1D_417570479/1D_417628766/1D_417764630/1D_417881766/1D_417902593/1D_417912644/1D_417953367/1D_417975493* | AA/GG/CC/CC/GG/AA/GG/CC | GG/AA//TT/TT/AA/GG/TT/TT | 184 | 110 |

Table S10 Genes nearby stable SNPs

| Trait | Marker | Annotation | Candidate gene | |
| --- | --- | --- | --- | --- |
|  |  |  | Former | Latter |
| Grain protein content | *6D_410156938* | intergenic | *TraesCS6D02G300600* | *TraesCS6D02G300700* |
|  | *2A_517926919* | intergenic | *TraesCS2A02G302300* | *TraesCS2A02G302400* |
|  | *3B_27229830* | intergenic | *TraesCS3B02G053300* | *TraesCS3B02G053400* |
|  | *5A_455461352* | intergenic | *TraesCS5A02G239200* | *TraesCS5A02G239300* |
| Flour protein content | *7A_647143478* | intergenic | *TraesCS7A02G453100* | *TraesCS7A02G453200* |
|  | *6D_410156938* | intergenic | *TraesCS6D02G300600* | *TraesCS6D02G300700* |
| Wet gluten content | *1D_412227592* | exonic | *TraesCS1D02G317400* | |
|  | *1D_415401424* | exonic | *TraesCS1D02G322339* | |
| Gluten index | *1D_411727551* | intergenic | *TraesCS1D02G316500* | *TraesCS1D02G316600* |
|  | *1D_412023762* | UTR3 | *TraesCS1D02G316900* | |
|  | *1D_415704212* | intergenic | *TraesCS1D02G323000* | *TraesCS1D02G323100* |
|  | *1D_415766966* | intergenic | *TraesCS1D02G323000* | *TraesCS1D02G323100* |
|  | *1D_411237031* | intergenic | *TraesCS1D02G316000* | *TraesCS1D02G316100* |
|  | *1D_411404496* | intergenic | *TraesCS1D02G316000* | *TraesCS1D02G316100* |
|  | *1D_412031073* | intergenic | *TraesCS1D02G317000* | *TraesCS1D02G317100* |
|  | *1D_412092560* | intergenic | *TraesCS1D02G317100* | *TraesCS1D02G317200* |
|  | *1D_412136490* | intergenic | *TraesCS1D02G317100* | *TraesCS1D02G317200* |
|  | *1D_412160361* | UTR5 | *TraesCS1D02G317200* | |
|  | *1D_412178540* | intergenic | *TraesCS1D02G317211* | *TraesCS1D02G317301* |
|  | *1D_412181832* | intergenic | *TraesCS1D02G317211* | *TraesCS1D02G317301* |
|  | *1D_412227592* | exonic | *TraesCS1D02G317400* | |
|  | *1D_413224542* | upstream | *TraesCS1D02G319000* | |
|  | *1D_413292355* | intergenic | *TraesCS1D02G319100* | *TraesCS1D02G319200* |
|  | *1D_413311188* | intergenic | *TraesCS1D02G319100* | *TraesCS1D02G319200* |
|  | *1D_413729079* | upstream | *TraesCS1D02G319700* | |
|  | *1D_413934525* | intergenic | *TraesCS1D02G320000* | *TraesCS1D02G320100* |
|  | *1D_414144652* | intergenic | *TraesCS1D02G320000* | *TraesCS1D02G320100* |
|  | *1D_414286643* | intronic | *TraesCS1D02G320200* | |
|  | *1D_414451522* | exonic | *TraesCS1D02G320500* | |
|  | *1D_414500837* | upstream | *TraesCS1D02G320800* | |
|  | *1D_414573947* | intergenic | *TraesCS1D02G321000* | *TraesCS1D02G321100* |
|  | *1D_414708956* | downstream | *TraesCS1D02G321100* | |
|  | *1D_415126157* | intergenic | *TraesCS1D02G321600* | *TraesCS1D02G321700* |
|  | *1D_415401424* | exonic | *TraesCS1D02G322339* | |
|  | *1D_415459431* | intergenic | *TraesCS1D02G322700* | *TraesCS1D02G322800* |
|  | *1D_415749624* | intergenic | *TraesCS1D02G323000* | *TraesCS1D02G323100* |
|  | *1D_416033546* | intergenic | *TraesCS1D02G323000* | *TraesCS1D02G323100* |
|  | *1D_416093683* | intergenic | *TraesCS1D02G323000* | *TraesCS1D02G323100* |
|  | *1D_416101167* | exonic | *TraesCS1D02G323100* | |
|  | *1D_416212526* | intergenic | *TraesCS1D02G323600* | *TraesCS1D02G323800* |
|  | *1D_411320325* | intergenic | *TraesCS1D02G316000* | *TraesCS1D02G316100* |
|  | *1D_411192168* | intergenic | *TraesCS1D02G315900* | *TraesCS1D02G316000* |
|  | *1D_415213648* | intergenic | *TraesCS1D02G321700* | *TraesCS1D02G321800* |
|  | *1B_559539573* | intergenic | *TraesCS1B02G332900* | *TraesCS1B02G333000* |
|  | *1A_20940731* | intergenic | *TraesCS1A02G037800* | *TraesCS1A02G037900* |
|  | *1B_561268092* | intergenic | *TraesCS1B02G334000* | *TraesCS1B02G334100* |
|  | *1D_411295558* | intergenic | *TraesCS1D02G316000* | *TraesCS1D02G316100* |
|  | *1D_411366902* | intergenic | *TraesCS1D02G316000* | *TraesCS1D02G316100* |
|  | *1D_411374953* | intergenic | *TraesCS1D02G316000* | *TraesCS1D02G316100* |
|  | *1D_412072331* | upstream | *TraesCS1D02G317100* | |
|  | *1D_412196822* | intergenic | *TraesCS1D02G317211* | *TraesCS1D02G317301* |
|  | *1D_412288344* | intergenic | *TraesCS1D02G317500* | *TraesCS1D02G317600* |
|  | *1D_412495297* | intergenic | *TraesCS1D02G317600* | *TraesCS1D02G317700* |
|  | *1D_414493743* | downstream | *TraesCS1D02G320700* | |
|  | *1D_415059649* | intergenic | *TraesCS1D02G321600* | *TraesCS1D02G321700* |
|  | *1D_415158238* | intergenic | *TraesCS1D02G321700* | *TraesCS1D02G321800* |
|  | *1D_415290059* | upstream | *TraesCS1D02G322100* | |
|  | *1D_415298532* | upstream | *TraesCS1D02G322200* | |
|  | *1D_415466562* | intergenic | *TraesCS1D02G322700* | *TraesCS1D02G322800* |
|  | *1D_415586977* | intergenic | *TraesCS1D02G322700* | *TraesCS1D02G322800* |
|  | *1D_415645580* | downstream | *TraesCS1D02G323000* | |
|  | *1D_416081310* | intergenic | *TraesCS1D02G323000* | *TraesCS1D02G323100* |
| Zeleny sedimentation value | *4D_238181951* | intergenic | *TraesCS4D02G160600* | *TraesCS4D02G160700* |
|  | *6B_370150478* | intergenic | *TraesCS6B02G230200* | *TraesCS6B02G230300* |
|  | *1A_225520951* | intergenic | *TraesCS1A02G137900* | *TraesCS1A02G138000* |
|  | *1A_9574254* | exonic | *TraesCS1A02G018800* | |
|  | *1A_21873794* | intergenic | *TraesCS1A02G039700* | *TraesCS1A02G039800* |
|  | *1A_236720351* | exonic | *TraesCS1A02G139500* | |
|  | *1B_53066005* | intergenic | *TraesCS1B02G068200* | *TraesCS1B02G068300* |
|  | *1D_232760* | ncRNA_exonic | *TraesCS1D02G000906* | |
|  | *1D_6299684* | upstream | *TraesCS1D02G011547* | |
|  | *4B_500743139* | intergenic | *TraesCS4B02G242000* | *TraesCS4B02G242100* |
|  | *1A_33855698* | intergenic | *TraesCS1A02G052000* | *TraesCS1A02G052100* |
|  | *1B_9992236* | exonic | *TraesCS1B02G021900* | |
|  | *1D_49579197* | intergenic | *TraesCS1D02G067708* | *TraesCS1D02G067700* |
|  | *2A_425759128* | intergenic | *TraesCS2A02G267000* | *TraesCS2A02G267100* |
|  | *4B_550381492* | intergenic | *TraesCS4B02G272400* | *TraesCS4B02G272500* |
|  | *6B_43515830* | intergenic | *TraesCS6B02G065700* | *TraesCS6B02G065749* |
|  | *7D_37949527* | intergenic | *TraesCS7D02G066900* | *TraesCS7D02G067100* |
|  | *1A_41516924* | stopgain | *TraesCS1A02G060400* | |
|  | *1B_67642598* | exonic | *TraesCS1B02G083700* | |
|  | *1A_96454293* | exonic | *TraesCS1A02G100200* | |
|  | *1D_56039301* | intergenic | *TraesCS1D02G074500* | *TraesCS1D02G074600* |
|  | *2A_192541883* | intergenic | *TraesCS2A02G209100* | *TraesCS2A02G209200* |
|  | *3A_686822185* | intergenic | *TraesCS3A02G446500* | *TraesCS3A02G446600* |
|  | *4A_177632748* | intergenic | *TraesCS4A02G132300* | *TraesCS4A02G132400* |
|  | *5B_400532969* | intergenic | *TraesCS5B02G225000* | *TraesCS5B02G225100* |
|  | *5D_408811401* | intergenic | *TraesCS5D02G313912* | *TraesCS5D02G314000* |
|  | *1A_12815998* | intergenic | *TraesCS1A02G027000* | *TraesCS1A02G027100* |
|  | *1A_31178918* | intergenic | *TraesCS1A02G049500* | *TraesCS1A02G049600* |
|  | *1A_508723612* | UTR5 | *TraesCS1A02G317300* | |
|  | *1D_19807137* | UTR5 | *TraesCS1D02G040548* | |
|  | *7D_96433791* | intergenic | *TraesCS7D02G148700* | *TraesCS7D02G148800* |
|  | *1A_34406785* | exonic | *TraesCS1A02G052300* | |
|  | *1A_291896187* | intergenic | *TraesCS1A02G162400* | *TraesCS1A02G162500* |
|  | *3A_65406649* | downstream | *TraesCS3A02G100700* | |
|  | *3B_28179353* | intergenic | *TraesCS3B02G054400* | *TraesCS3B02G054500* |

Table S11 Annotation of candidate genes

| ID | Start | End | Annotation |
| --- | --- | --- | --- |
| *TraesCS6B02G230300.2* | 371610328 | 371612158 | 60S ribosomal protein L6 |
| *TraesCS1A02G137700.2* | 223937889 | 223949190 | Tobamovirus multiplication protein 2A |
| *TraesCS1B02G068200.1* | 53050199 | 53052675 | Co-chaperone protein p23 |
| *TraesCS1B02G068500.2* | 53246153 | 53249027 | Rhodopsin |
| *TraesCS1D02G000700.1* | 209608 | 210213 | Gliadin/avenin-like seed protein |
| *TraesCS1D02G000800.2* | 216466 | 219484 | Peptidyl-prolyl cis-trans isomerase · Gene: CYP20-1 |
| *TraesCS1D02G001200.1* | 321910 | 322893 | Gamma gliadin-D2 |
| *TraesCS1D02G001300.1* | 339837 | 340811 | Delta gliadin-D1 |
| *TraesCS1D02G009400.1* | 5139131 | 5140186 | —— |
| *TraesCS1D02G018300.1* | 7918701 | 7921587 | V-type proton ATPase proteolipid subunit |
| *TraesCS1B02G286800.1* | 498859326 | 498861029 | rRNA N-glycosidase |
| *TRAESCS1D02G323100* | 416098486 | 416102366 | H/ACA ribonucleoprotein complex non-core subunit NAF1 |
| *TraesCS1A02G100200* | 96453203 | 96455187 | F-box domain-containing protein |

Table S12 Significant SNPs associated with protein quality traits identified in individual environment by genome-wide association study

| Trait | Environment | Marker | Chr | Pos | *P* |
| --- | --- | --- | --- | --- | --- |
| Grain protein content | 2020EM | *3D_185798059* | 3D | 185798059 | 8.02E-05 |
|  | 2020QT | *3B_280825292* | 3B | 280825292 | 6.32E-05 |
|  |  | *6D_410156938* | 6D | 410156938 | 9.41E-05 |
|  | 2021EM | *1A_193798375* | 1A | 193798375 | 4.31E-05 |
|  |  | *2A_517926919* | 2A | 517926919 | 7.79E-05 |
|  |  | *2B_158150159* | 2B | 158150159 | 7.43E-05 |
|  |  | *3B_27229830* | 3B | 27229830 | 1.29E-05 |
|  |  | *4A_596897908* | 4A | 596897908 | 7.50E-05 |
|  |  | *5A_455119498* | 5A | 455119498 | 8.22E-05 |
|  |  | *5A_455204777* | 5A | 455204777 | 4.18E-05 |
|  |  | *5A_455342738* | 5A | 455342738 | 4.45E-05 |
|  |  | *5A_455461352* | 5A | 455461352 | 3.54E-05 |
|  |  | *5A_456970795* | 5A | 456970795 | 8.89E-05 |
|  |  | *6D_418313887* | 6D | 418313887 | 2.97E-05 |
|  |  | *7D_629293059* | 7D | 629293059 | 9.83E-05 |
|  | 2021QT | *5B_587603115* | 5B | 587603115 | 6.00E-05 |
| Flour protein content | 2020EM | *6D_333490747* | 6D | 333490747 | 5.6279E-05 |
|  |  | *7A_647143478* | 7A | 647143478 | 8.7676E-05 |
|  | 2020QT | *5A_455204777* | 5A | 455204777 | 0.0000999 |
|  |  | *6D_410156938* | 6D | 410156938 | 0.000044 |
|  | 2021EM | *1B_634512089* | 1B | 634512089 | 0.0000633 |
|  |  | *3D_58370641* | 3D | 58370641 | 0.0000755 |
|  |  | *6A_543213608* | 6A | 543213608 | 0.0000476 |
|  | 2021QT | *1A_460844585* | 1A | 460844585 | 7.8918E-05 |
|  |  | *5A_615827839* | 5A | 615827839 | 9.8657E-05 |
| Wet gluten content | 2020EM | *2B_585851234* | 2B | 585851234 | 2.27E-05 |
|  |  | *5B_538720257* | 5B | 538720257 | 3.42E-05 |
|  | 2020QT | *1D_412227592* | 1D | 412227592 | 8.46E-05 |
|  |  | *1D_415401424* | 1D | 415401424 | 3.65E-05 |
|  |  | *2B_708663362* | 2B | 708663362 | 3.35E-05 |
|  |  | *3D_231453854* | 3D | 231453854 | 4.69E-05 |
|  |  | *4B_37727016* | 4B | 37727016 | 9.23E-05 |
|  |  | *7A_647143478* | 7A | 647143478 | 7.48E-05 |
|  | 2021EM | *2A_728416651* | 2A | 728416651 | 3.25E-05 |
|  |  | *2A_746728243* | 2A | 746728243 | 7.60E-06 |
|  |  | *4B_12626397* | 4B | 12626397 | 8.34E-05 |
|  |  | *4B_599260253* | 4B | 599260253 | 3.53E-05 |
|  |  | *4B_602536647* | 4B | 602536647 | 1.49E-05 |
|  |  | *4B_603575880* | 4B | 603575880 | 2.76E-05 |
|  |  | *4B_662778982* | 4B | 662778982 | 5.22E-05 |
|  |  | *4D_159989855* | 4D | 159989855 | 5.41E-05 |
|  |  | *5A_464484237* | 5A | 464484237 | 8.01E-05 |
|  |  | *5A_467375467* | 5A | 467375467 | 8.40E-05 |
|  |  | *5A_469515528* | 5A | 469515528 | 7.38E-05 |
| Gluten index | 2020EM | *1B_559539573* | 1B | 559539573 | 3.57E-05 |
|  |  | *1D_411192168* | 1D | 411192168 | 9.68E-05 |
|  |  | *1D_411320325* | 1D | 411320325 | 1.68E-05 |
|  |  | *1D_411727551* | 1D | 411727551 | 7.57E-05 |
|  |  | *1D_412023762* | 1D | 412023762 | 5.85E-05 |
|  |  | *1D_415213648* | 1D | 415213648 | 6.50E-05 |
|  |  | *1D_415704212* | 1D | 415704212 | 1.43E-05 |
|  |  | *1D_415766966* | 1D | 415766966 | 4.12E-05 |
|  | 2020QT | *1A_20940731* | 1A | 20940731 | 6.18E-05 |
|  |  | *1B_561268092* | 1B | 561268092 | 6.10E-05 |
|  |  | *1D_411192168* | 1D | 411192168 | 2.83E-05 |
|  |  | *1D_411237031* | 1D | 411237031 | 1.95E-05 |
|  |  | *1D_411295558* | 1D | 411295558 | 5.86E-05 |
|  |  | *1D_411366902* | 1D | 411366902 | 8.32E-05 |
|  |  | *1D_411374953* | 1D | 411374953 | 2.17E-05 |
|  |  | *1D_411404496* | 1D | 411404496 | 1.95E-05 |
|  |  | *1D_411727551* | 1D | 411727551 | 2.25E-05 |
|  |  | *1D_412023762* | 1D | 412023762 | 6.24E-05 |
|  |  | *1D_412031073* | 1D | 412031073 | 1.19E-05 |
|  |  | *1D_412072331* | 1D | 412072331 | 6.11E-06 |
|  |  | *1D_412092560* | 1D | 412092560 | 1.00E-05 |
|  |  | *1D_412136490* | 1D | 412136490 | 8.30E-06 |
|  |  | *1D_412160361* | 1D | 412160361 | 4.19E-06 |
|  |  | *1D_412178540* | 1D | 412178540 | 2.62E-05 |
|  |  | *1D_412181832* | 1D | 412181832 | 7.98E-05 |
|  |  | *1D_412196822* | 1D | 412196822 | 1.43E-05 |
|  |  | *1D_412227592* | 1D | 412227592 | 7.28E-07 |
|  |  | *1D_412288344* | 1D | 412288344 | 1.51E-05 |
|  |  | *1D_412495297* | 1D | 412495297 | 1.37E-05 |
|  |  | *1D_413224542* | 1D | 413224542 | 4.52E-07 |
|  |  | *1D_413292355* | 1D | 413292355 | 3.63E-06 |
|  |  | *1D_413311188* | 1D | 413311188 | 2.51E-05 |
|  |  | *1D_413729079* | 1D | 413729079 | 3.63E-06 |
|  |  | *1D_413934525* | 1D | 413934525 | 2.09E-05 |
|  |  | *1D_414144652* | 1D | 414144652 | 1.58E-05 |
|  |  | *1D_414286643* | 1D | 414286643 | 1.17E-05 |
|  |  | *1D_414451522* | 1D | 414451522 | 2.43E-05 |
|  |  | *1D_414493743* | 1D | 414493743 | 7.55E-06 |
|  |  | *1D_414500837* | 1D | 414500837 | 4.17E-06 |
|  |  | *1D_414573947* | 1D | 414573947 | 4.27E-06 |
|  |  | *1D_414708956* | 1D | 414708956 | 4.28E-06 |
|  |  | *1D_415059649* | 1D | 415059649 | 1.34E-05 |
|  |  | *1D_415126157* | 1D | 415126157 | 5.20E-06 |
|  |  | *1D_415158238* | 1D | 415158238 | 5.11E-05 |
|  |  | *1D_415213648* | 1D | 415213648 | 1.12E-05 |
|  |  | *1D_415267964* | 1D | 415267964 | 8.70E-05 |
|  |  | *1D_415290059* | 1D | 415290059 | 7.29E-06 |
|  |  | *1D_415298532* | 1D | 415298532 | 2.31E-06 |
|  |  | *1D_415401424* | 1D | 415401424 | 2.37E-06 |
|  |  | *1D_415459431* | 1D | 415459431 | 2.01E-05 |
|  |  | *1D_415466562* | 1D | 415466562 | 1.10E-05 |
|  |  | *1D_415586977* | 1D | 415586977 | 6.00E-06 |
|  |  | *1D_415645580* | 1D | 415645580 | 4.56E-06 |
|  |  | *1D_415704212* | 1D | 415704212 | 1.08E-05 |
|  |  | *1D_415749624* | 1D | 415749624 | 2.19E-05 |
|  |  | *1D_415766966* | 1D | 415766966 | 5.02E-07 |
|  |  | *1D_416033546* | 1D | 416033546 | 4.26E-06 |
|  |  | *1D_416081310* | 1D | 416081310 | 7.17E-06 |
|  |  | *1D_416093683* | 1D | 416093683 | 4.58E-06 |
|  |  | *1D_416101167* | 1D | 416101167 | 5.41E-05 |
|  |  | *1D_416212526* | 1D | 416212526 | 2.76E-05 |
|  |  | *1D_416282099* | 1D | 416282099 | 9.90E-05 |
|  |  | *3A_724651078* | 3A | 724651078 | 6.96E-05 |
|  |  | *3B_556713931* | 3B | 556713931 | 9.03E-05 |
|  |  | *4B_37764579* | 4B | 37764579 | 4.53E-05 |
|  |  | *5B_655916574* | 5B | 655916574 | 4.52E-05 |
|  | 2021EM | *1D_411237031* | 1D | 411237031 | 6.96E-05 |
|  |  | *1D_411320325* | 1D | 411320325 | 9.41E-05 |
|  |  | *1D_411404496* | 1D | 411404496 | 3.78E-05 |
|  |  | *1D_411727551* | 1D | 411727551 | 9.99E-06 |
|  |  | *1D_412023762* | 1D | 412023762 | 9.15E-06 |
|  |  | *1D_412031073* | 1D | 412031073 | 8.50E-05 |
|  |  | *1D_412092560* | 1D | 412092560 | 1.51E-05 |
|  |  | *1D_412136490* | 1D | 412136490 | 4.46E-05 |
|  |  | *1D_412160361* | 1D | 412160361 | 4.09E-05 |
|  |  | *1D_412178540* | 1D | 412178540 | 5.34E-05 |
|  |  | *1D_412181832* | 1D | 412181832 | 6.98E-05 |
|  |  | *1D_412227592* | 1D | 412227592 | 2.73E-05 |
|  |  | *1D_413224542* | 1D | 413224542 | 3.32E-05 |
|  |  | *1D_413292355* | 1D | 413292355 | 6.04E-05 |
|  |  | *1D_413311188* | 1D | 413311188 | 3.62E-05 |
|  |  | *1D_413729079* | 1D | 413729079 | 5.87E-05 |
|  |  | *1D_413934525* | 1D | 413934525 | 1.81E-05 |
|  |  | *1D_414144652* | 1D | 414144652 | 6.27E-05 |
|  |  | *1D_414286643* | 1D | 414286643 | 4.99E-05 |
|  |  | *1D_414451522* | 1D | 414451522 | 3.64E-05 |
|  |  | *1D_414500837* | 1D | 414500837 | 5.99E-05 |
|  |  | *1D_414573947* | 1D | 414573947 | 5.45E-05 |
|  |  | *1D_414708956* | 1D | 414708956 | 5.92E-05 |
|  |  | *1D_415126157* | 1D | 415126157 | 3.47E-05 |
|  |  | *1D_415401424* | 1D | 415401424 | 3.96E-05 |
|  |  | *1D_415459431* | 1D | 415459431 | 1.68E-05 |
|  |  | *1D_415704212* | 1D | 415704212 | 2.36E-05 |
|  |  | *1D_415749624* | 1D | 415749624 | 2.29E-05 |
|  |  | *1D_415766966* | 1D | 415766966 | 2.10E-05 |
|  |  | *1D_416033546* | 1D | 416033546 | 6.01E-05 |
|  |  | *1D_416093683* | 1D | 416093683 | 3.44E-05 |
|  |  | *1D_416101167* | 1D | 416101167 | 3.32E-06 |
|  |  | *1D_416212526* | 1D | 416212526 | 3.71E-05 |
|  |  | *6D_472567591* | 6D | 472567591 | 4.11E-05 |
|  | 2021QT | *2A_65950236* | 2A | 65950236 | 6.08E-05 |
|  |  | *3B_595991536* | 3B | 595991536 | 7.64E-05 |
|  |  | *3B_596418370* | 3B | 596418370 | 5.09E-05 |
|  |  | *3B_596598146* | 3B | 596598146 | 9.01E-05 |
|  |  | *3B_596632634* | 3B | 596632634 | 2.91E-05 |
|  |  | *4D_238181951* | 4D | 238181951 | 8.11E-05 |
|  |  | *5B_566591669* | 5B | 566591669 | 2.73E-05 |
|  |  | *5B_569553692* | 5B | 569553692 | 8.91E-05 |
|  |  | *6A_76838492* | 6A | 76838492 | 1.94E-07 |
| Zeleny sedimentation value | 2020EM | *1A_9574254* | 1A | 9574254 | 2.72E-05 |
|  |  | *1A_21873794* | 1A | 21873794 | 4.41E-06 |
|  |  | *1A_33855698* | 1A | 33855698 | 8.59E-05 |
|  |  | *1A_34406785* | 1A | 34406785 | 3.95E-05 |
|  |  | *1A_41516924* | 1A | 41516924 | 1.34E-05 |
|  |  | *1A_96454293* | 1A | 96454293 | 2.64E-05 |
|  |  | *1A_225520951* | 1A | 225520951 | 8.86E-05 |
|  |  | *1A_236720351* | 1A | 236720351 | 7.84E-09 |
|  |  | *1A_291896187* | 1A | 291896187 | 6.13E-05 |
|  |  | *1B_9992236* | 1B | 9992236 | 4.66E-06 |
|  |  | *1B_50124992* | 1B | 50124992 | 7.59E-05 |
|  |  | *1B_53066005* | 1B | 53066005 | 2.69E-06 |
|  |  | *1B_67642598* | 1B | 67642598 | 1.16E-05 |
|  |  | *1B_109213613* | 1B | 109213613 | 3.10E-05 |
|  |  | *1B_152959732* | 1B | 152959732 | 5.10E-05 |
|  |  | *1B_288359181* | 1B | 288359181 | 9.53E-05 |
|  |  | *1B_329613018* | 1B | 329613018 | 1.89E-05 |
|  |  | *1D_232760* | 1D | 232760 | 3.80E-05 |
|  |  | *1D_6299684* | 1D | 6299684 | 2.33E-05 |
|  |  | *1D_49579197* | 1D | 49579197 | 4.55E-05 |
|  |  | *1D_56039301* | 1D | 56039301 | 9.44E-05 |
|  |  | *1D_57947466* | 1D | 57947466 | 6.24E-05 |
|  |  | *2A_192541883* | 2A | 192541883 | 4.68E-05 |
|  |  | *2A_425759128* | 2A | 425759128 | 2.12E-05 |
|  |  | *3A_65406649* | 3A | 65406649 | 7.08E-05 |
|  |  | *3A_686822185* | 3A | 686822185 | 4.36E-05 |
|  |  | *4A_98564308* | 4A | 98564308 | 8.98E-05 |
|  |  | *4A_177632748* | 4A | 177632748 | 9.35E-06 |
|  |  | *4A_597351177* | 4A | 597351177 | 2.44E-06 |
|  |  | *4B_500743139* | 4B | 500743139 | 1.54E-06 |
|  |  | *4B_550381492* | 4B | 550381492 | 3.92E-05 |
|  |  | *4D_238181951* | 4D | 238181951 | 5.66E-07 |
|  |  | *5A_74538453* | 5A | 74538453 | 6.82E-05 |
|  |  | *5B_194081612* | 5B | 194081612 | 3.73E-05 |
|  |  | *5B_400532969* | 5B | 400532969 | 3.69E-05 |
|  |  | *5D_408811401* | 5D | 408811401 | 3.10E-06 |
|  |  | *6B_43515830* | 6B | 43515830 | 6.46E-05 |
|  |  | *6B_370150478* | 6B | 370150478 | 3.16E-06 |
|  |  | *7D_19103399* | 7D | 19103399 | 2.00E-05 |
|  |  | *7D_37949527* | 7D | 37949527 | 6.82E-06 |
|  |  | *7D_490578388* | 7D | 490578388 | 7.67E-05 |
|  | 2020QT | *1A_225520951* | 1A | 225520951 | 5.75E-05 |
|  |  | *4D_238181951* | 4D | 238181951 | 1.65E-05 |
|  |  | *6A_24240974* | 6A | 24240974 | 3.53E-05 |
|  |  | *6B_370150478* | 6B | 370150478 | 3.91E-05 |
|  | 2021EM | *1A_9574254* | 1A | 9574254 | 1.46E-05 |
|  |  | *1A_21873794* | 1A | 21873794 | 7.54E-05 |
|  |  | *1A_41516924* | 1A | 41516924 | 5.97E-05 |
|  |  | *1A_236720351* | 1A | 236720351 | 7.17E-06 |
|  |  | *1B_53066005* | 1B | 53066005 | 3.06E-05 |
|  |  | *1B_67642598* | 1B | 67642598 | 8.64E-05 |
|  |  | *1D_232760* | 1D | 232760 | 6.07E-05 |
|  |  | *1D_6299684* | 1D | 6299684 | 9.61E-05 |
|  |  | *2D_92135923* | 2D | 92135923 | 7.20E-05 |
|  |  | *3B_28179353* | 3B | 28179353 | 3.36E-05 |
|  |  | *4A_211670106* | 4A | 211670106 | 2.04E-05 |
|  |  | *4B_500743139* | 4B | 500743139 | 1.30E-05 |
|  |  | *4D_238181951* | 4D | 238181951 | 7.22E-06 |
|  |  | *6B_370150478* | 6B | 370150478 | 8.70E-05 |
|  | 2021QT | *1A_9574254* | 1A | 9574254 | 3.00E-06 |
|  |  | *1A_12815998* | 1A | 12815998 | 2.45E-06 |
|  |  | *1A_21873794* | 1A | 21873794 | 3.75E-05 |
|  |  | *1A_27754704* | 1A | 27754704 | 3.95E-05 |
|  |  | *1A_31178918* | 1A | 31178918 | 5.29E-05 |
|  |  | *1A_33855698* | 1A | 33855698 | 9.06E-05 |
|  |  | *1A_34406785* | 1A | 34406785 | 5.83E-05 |
|  |  | *1A_225520951* | 1A | 225520951 | 3.69E-06 |
|  |  | *1A_236720351* | 1A | 236720351 | 3.00E-07 |
|  |  | *1A_291896187* | 1A | 291896187 | 4.29E-05 |
|  |  | *1A_508723612* | 1A | 508723612 | 4.52E-05 |
|  |  | *1A_510672068* | 1A | 510672068 | 2.38E-05 |
|  |  | *1A_512466974* | 1A | 512466974 | 1.13E-05 |
|  |  | *1B_9992236* | 1B | 9992236 | 4.99E-07 |
|  |  | *1B_53066005* | 1B | 53066005 | 1.10E-06 |
|  |  | *1B_560217196* | 1B | 560217196 | 9.03E-05 |
|  |  | *1B_613410943* | 1B | 613410943 | 5.11E-05 |
|  |  | *1B_614184221* | 1B | 614184221 | 9.30E-05 |
|  |  | *1B_657973425* | 1B | 657973425 | 4.84E-05 |
|  |  | *1D_232760* | 1D | 232760 | 9.72E-06 |
|  |  | *1D_6299684* | 1D | 6299684 | 3.36E-05 |
|  |  | *1D_19807137* | 1D | 19807137 | 2.55E-05 |
|  |  | *1D_49579197* | 1D | 49579197 | 8.16E-05 |
|  |  | *2A_425759128* | 2A | 425759128 | 7.22E-06 |
|  |  | *2A_610045334* | 2A | 610045334 | 5.99E-06 |
|  |  | *2D_439444513* | 2D | 439444513 | 4.08E-05 |
|  |  | *3A_65406649* | 3A | 65406649 | 8.49E-05 |
|  |  | *3B_28179353* | 3B | 28179353 | 8.86E-05 |
|  |  | *3B_773524426* | 3B | 773524426 | 2.29E-05 |
|  |  | *4A_632989028* | 4A | 632989028 | 1.84E-05 |
|  |  | *4A_633310601* | 4A | 633310601 | 8.30E-06 |
|  |  | *4A_634367853* | 4A | 634367853 | 7.34E-06 |
|  |  | *4A_635367530* | 4A | 635367530 | 9.63E-05 |
|  |  | *4B_500743139* | 4B | 500743139 | 4.62E-05 |
|  |  | *4B_550381492* | 4B | 550381492 | 6.87E-06 |
|  |  | *4D_238181951* | 4D | 238181951 | 1.24E-07 |
|  |  | *4D_413760613* | 4D | 413760613 | 6.79E-05 |
|  |  | *5A_488261672* | 5A | 488261672 | 9.73E-05 |
|  |  | *5B_657385403* | 5B | 657385403 | 5.71E-05 |
|  |  | *6B_43515830* | 6B | 43515830 | 4.22E-05 |
|  |  | *6B_370150478* | 6B | 370150478 | 1.53E-07 |
|  |  | *6D_110316095* | 6D | 110316095 | 1.97E-05 |
|  |  | *7D_37949527* | 7D | 37949527 | 8.16E-06 |
|  |  | *7D_89106717* | 7D | 89106717 | 7.21E-05 |
|  |  | *7D_96433791* | 7D | 96433791 | 4.94E-07 |


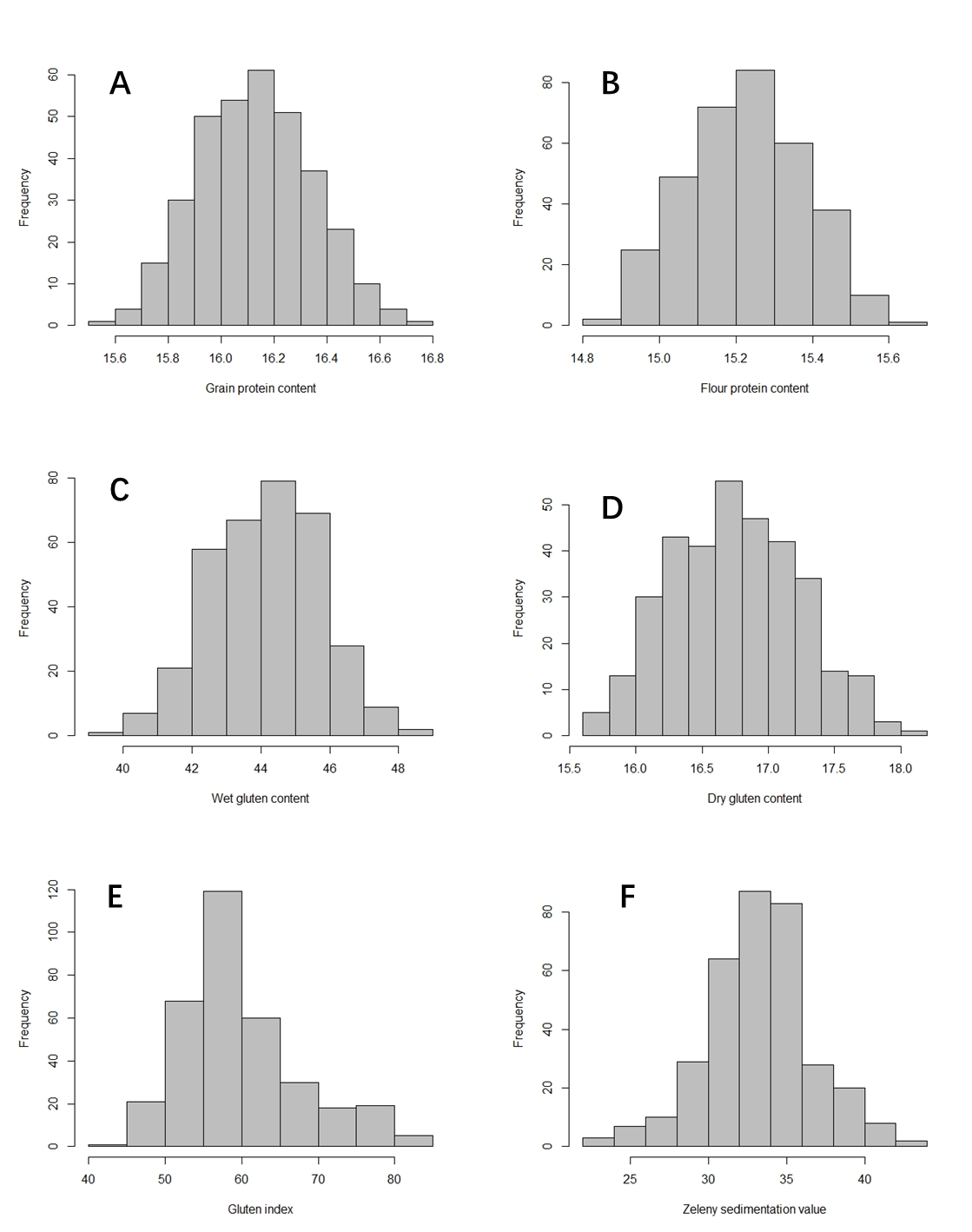


Figure S1 Frequency distributions of BLUP values for protein parameters in 341 winter wheat genotypes.


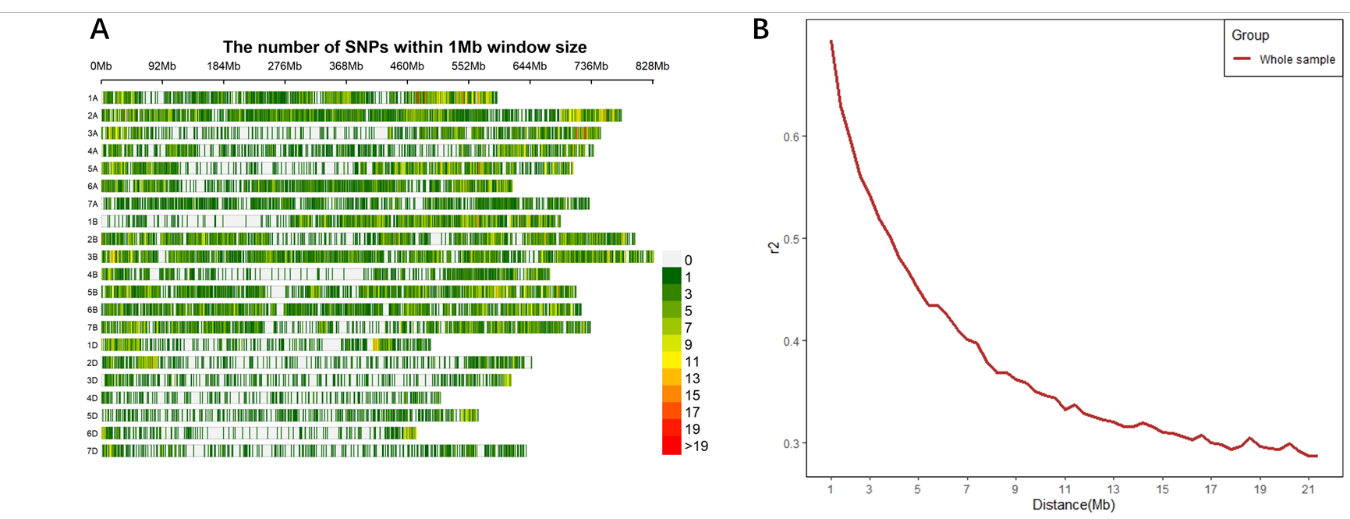
 Figure S2 Distribution of SNPs on chromosomes (A) and LD of the association panel (B) in whole chromosome.


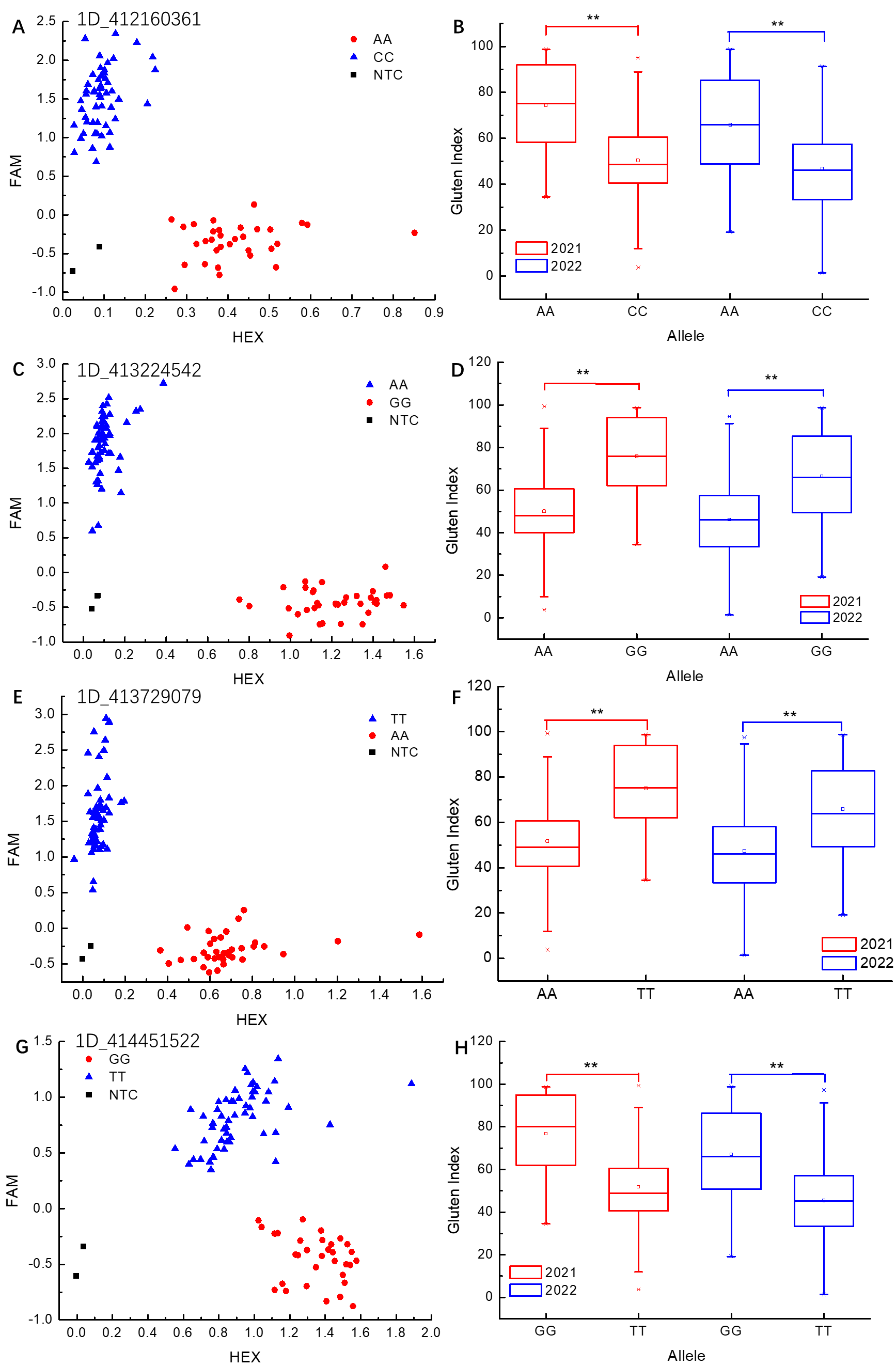


Figure S3 Kompetitive allele-specific PCR (KASP) verification of a significant single nucleotide polymorphism (SNP) related to the gluten index. A, C, E, G, scatter plots of KASP markers; B, D, F, H, the variance of gluten index for accessions with different alleles; Red dots and blue triangles represent the homozygous genotypes, while the green diamonds represent heterozygous genotypes; The black squares on the bottom left of the plot indicate the no-template control.


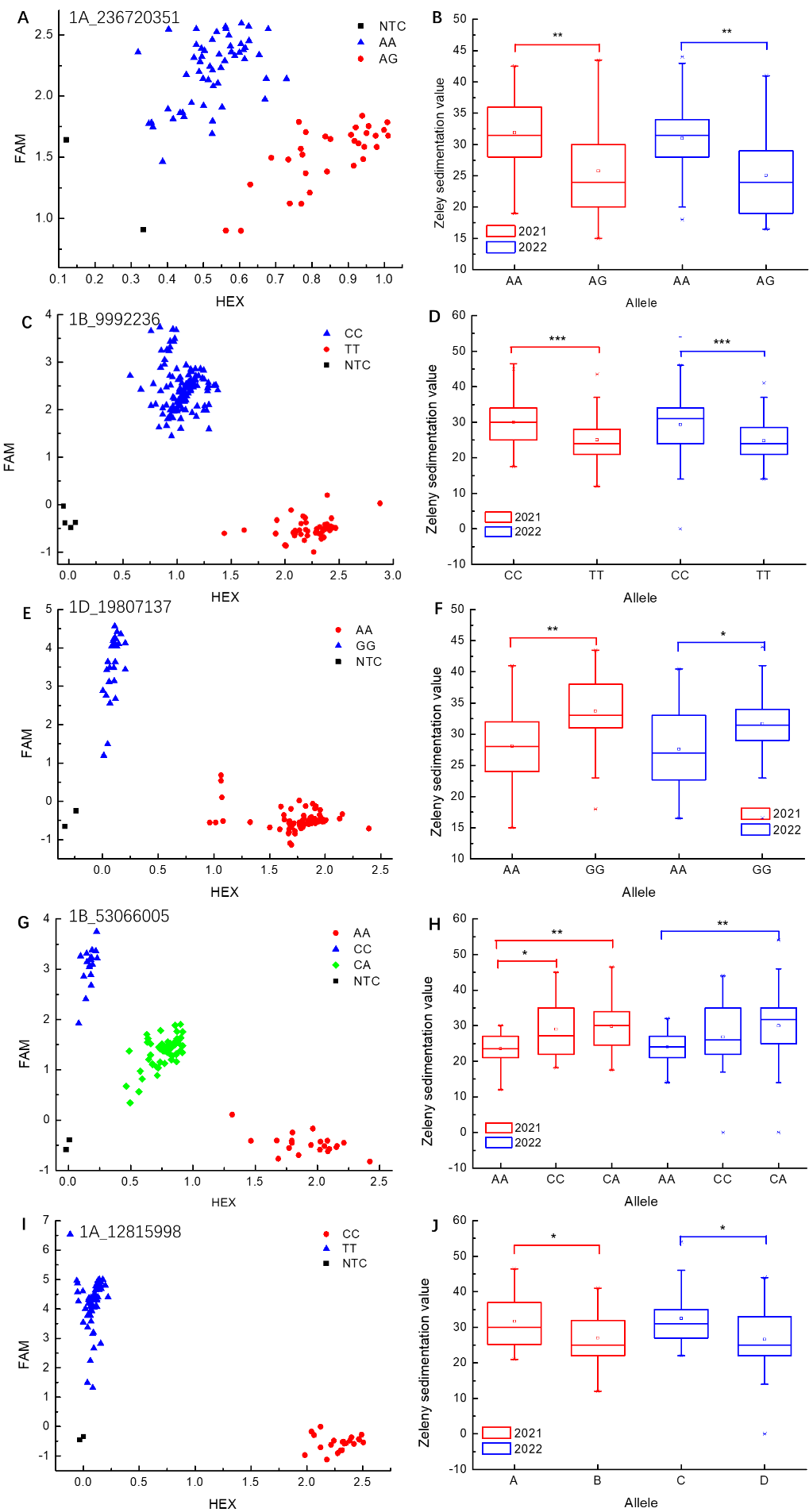


Figure S4 Kompetitive allele-specific PCR (KASP) verification of a significant single nucleotide polymorphism (SNP) associated with Zeleny sedimentation value. A, C, E, G, I, scatter plots for KASP markers; B, D, F, H, J, the variance of g Zeleny sedimentation value for accessions with different alleles. The red dots and blue triangles represent the homozygous genotypes, while the green diamonds represent the heterozygous genotypes; The black squares on the bottom left of the plot indicate the no-template control.


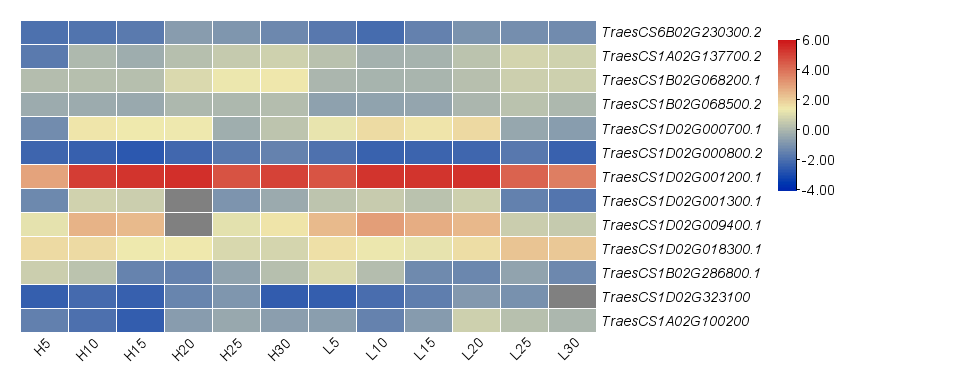


Figure S5 Heat map of candidate genes for protein quality traits. "H" refers to Hongzhitou, which has a high protein content; "L" refers to Huaimai0208, which has a low protein content; 5, 10, 15, 20, 25, and 30 refer to the number of days after anthesis.
